# Co-administration of Paediatric Medicines with Food and Drinks in the Context of Their Physicochemical Properties—a Global Perspective on Practices and Recommendations

**DOI:** 10.1208/s12248-020-0432-9

**Published:** 2020-03-04

**Authors:** Joana Martir, Talia Flanagan, James Mann, Nikoletta Fotaki

**Affiliations:** 1 Department of Pharmacy and Pharmacology, University of Bath, Claverton Down, Bath, BA2 7AY UK; 2 Pharmaceutical Technology and Development, Astra Zeneca, Macclesfield, UK; 3 Currently at UCB Pharma, Chemin du Foriest, B - 1420 Braine-l’Alleud, Belgium

**Keywords:** Paediatrics, Drug manipulation, Food and drinks, Formulation, Biopharmaceutical and physicochemical properties

## Abstract

Medicine co-administration with food or drink vehicles is a common administration practice in paediatrics. The aims of this review were (i) to describe the current recommended strategies for co-administration of paediatric medicines with food and drinks (vehicles); (ii) to compare current administration recommendations from different countries; and (iii) to obtain a global perspective on the rationale behind the choice of recommended vehicle, in the context of the physicochemical properties of the drug and formulation. This study used a defined search strategy on the practices of paediatric medicine co-administration with vehicles, recommended in a commonly used paediatric and neonatal handbook, in addition to the information previously gathered from UK formularies. Logistic regression analysis was performed to further understand the biopharmaceutical basis of the choice of recommended vehicle for medicine co-administration. Differences were identified in the type of vehicles globally recommended for medicine co-administration. Ultimately, a statistical model was developed which provided an understanding on which vehicle is recommended for use with drugs/formulations, with basis on their biopharmaceutical properties. Overall, this review highlights the areas where further information is needed to support standardised procedures and guide the recommendation of age-appropriate and acceptable vehicles for use in the co-administration of paediatric medicines. Unified requirements are needed for harmonisation of the practice of medicine co-administration with vehicles. *In vitro* and/or *in silico* tools should be developed to evaluate the potential clinical outcomes of this practice during paediatric drug development.

**Electronic supplementary material:**

The online version of this article (10.1208/s12248-020-0432-9) contains supplementary material, which is available to authorized users.

## INTRODUCTION

A shift has been observed towards the development of user-friendly, preservative-free, taste-masked formulations (e.g. multiparticulate single-use solid dosage forms) for the paediatric population [1–4]. However, several factors hinder paediatric drug product development, such as the heterogeneity of the paediatric population, the knowledge gaps in the understanding of developmental changes in physiology and organ maturation, the need for outcome measures for paediatric patients, the parental involvement, the ethical and economic constraints and the adaptations of required research procedures and settings to accommodate paediatric anatomic/cognitive development [2, 3]. Consequently, lack of medicines designed and studied for use in paediatrics is still an issue, and in many therapeutic areas the need for authorised paediatric formulations remains [2]. When age-appropriate licensed formulations are not available, there are several options for providing paediatric patients with suitable treatments. These include: (i) seeking a licensed therapeutic alternative, (ii) importing products authorised in other countries (which can be costly, time-consuming, and often subject to strict regulations), (iii) compounding medicines within the pharmacy (i.e. preparing an unlicensed medicine to meet specific patient needs) or (iv) manipulating licensed dosage forms [5–7].

Drug manipulation is a widely spread, common practice for drug administration and refers to handling of medicines to make them suitable for intended administration, for example when a specific dose not available is needed, to improve taste and/or patient acceptability and compliance [5, 8]. Examples of medicine manipulation include dividing/crushing a tablet, opening a capsule and emptying its contents, making serial dilutions, mixing syrup into a crushed tablet to prepare an extemporaneous preparation, and mixing a medicine with food or drinks (vehicles) to aid administration. Several risks have been associated with drug manipulation practices, including inconsistent results in terms of dose accuracy and possible effects on drug stability, solubility and bioavailability [7, 9–11]. Ultimately, these practices may lead to subtherapeutic or even toxic drug levels and/or increase the risk of side effects, which raises safety concerns [1, 7, 12, 13]. Therefore, there is a need to evaluate the impact of drug manipulation practices and standardise recommendations and administration procedures to reduce the risks associated with medicine manipulation.

The most practiced manipulation technique to facilitate paediatric administration is to mix a dosage form with vehicles [13, 14]. Small amounts of food or drinks can be used as vehicles for oral administration of medicines, provided they do not alter formulation performance, and are compatible and suitable for use in the targeted patient age group [5, 8, 15]. Therefore, when this practice is intended, assessment of quality attributes of the mixture formulation-vehicle should be performed (e.g. potency assay, and *in vitro* dissolution/release studies) [5, 15].

Clear instructions on the optional use of vehicles to facilitate medicine administration should be included in the labelling, summary of product characteristics (SmPC) and patient information leaflet (PIL) of the commercial formulation [5, 15]. However, many factors such as seasonal, regional and climate conditions as well as age-related characteristics, disease state and indication will influence vehicle composition or preference, respectively [7]. For example, diet preferences will change depending on the age group (e.g. younger age groups have mostly a liquid diet and so mixing with a solid food would not be an option), country and physiologic characteristics (e.g. swallowability problems in very young ages) [6]. Vehicle selection will also be influenced by therapeutic needs; e.g. the vehicle to be selected for administration of an oral antiepileptic to a seizing child should allow a fast onset of drug action whilst targeting the swallowing issues presented as the disease symptoms. Thus, the best candidates for use in practice are vehicles with relatively small fluctuations in their macronutrient composition and physicochemical characteristics, such as vehicle viscosity and pH, and binding/chelation characteristics. Moreover, vehicle candidates should be screened concerning their interaction with drug and formulation properties and their adequacy to the target age group [5, 15].

To standardise quality and availability of paediatric medicines, national and global initiatives have been undertaken. In the UK, the Manipulation Of Drugs In Children (MODRIC) guidelines have been developed to provide information for healthcare professionals (primarily), parents and carers; these guidelines summarise the evidence from a systematic review as well as the findings from an observational study of manipulations in neonatal and paediatric practice, and a questionnaire administered to a sample of paediatric nurses throughout the UK. These guidelines aim to (i) describe options available to avoid manipulation of medicines, (ii) provide readily accessible, easy to read guidance for delivering appropriate and reproducible medicine doses, where manipulation is necessary, (iii) inform best practices and potential risks associated with manipulation of medicines for the patient, product and operator, and (iv) raise awareness amongst regulators, advisory bodies and the pharmaceutical industry that manipulations occur but should be standardised [16]. In the EU, the European Committee on Pharmaceuticals and Pharmaceutical Care (CD-P-PH) and the European Directorate for the Quality of Medicines & HealthCare (EDQM) have recently launched an initiative towards the compilation of a pan-European Paediatric Formulary, consisting of monographs for extemporaneous formulations, based on national or regional information [17, 18]. This formulary is intended to give indications on the preparation of extemporaneous formulations for paediatric medicines and harmonise medicine administration practices. It should be noted though that information regarding formulation co-administration with food and drinks is not included in the pilot monographs available [17, 19]. In practice, recent studies have shown that medicine co-administration with vehicles is often performed without following recommended procedures [7]. Parents, carers and healthcare professionals often choose or let the child choose the food or drink used for medicine co-administration, without following the recommendations stated on the PIL or SmPC of the medicine [7, 13, 14]. The implications of the uninformed use of vehicles for medicine co-administration on drug safety and efficacy are often not taken into consideration.

Recommendations for mixing oral drugs with vehicles for paediatric administration, as described in national and hospital formularies from the UK, have been recently reviewed [7]. Differences in the type of vehicles recommended and those used in current practice were identified, and it was revealed that vehicle recommendations are made on a case-by-case basis, without a clear scientific rationale behind the choice of vehicle and/or depending on the patient and/or administration setting (e.g. hospital or home). The importance of considering the possible physicochemical or bioavailability changes that may occur from the co-administration of medicines with vehicles in the paediatric population was highlighted.

Several other studies have aimed to identify the prevalence of drug manipulation practices and understand the problems experienced in both outpatient and clinical settings, including practices of medicine co-administration with food and drinks [20, 21]. However, several gaps remain, including the lack of an understanding of which vehicles are used for medicine co-administration in different counties and an understanding behind the choice of the recommended vehicle, in the context of the physicochemical properties of the drug and formulation.

In this review, the vehicles currently recommended to be used for medicine co-administration to paediatric patients are discussed on a global perspective. Firstly, vehicle recommendations as reported in a paediatric handbook frequently used in clinical practice (in the USA and other countries) were compared with previously gathered information from other formularies. Secondly, differences between recommendations were evaluated. Similarly to our previous review [7], the types of vehicles recommended to be mixed with medicines were correlated to the type of formulation and the BCS class of the drug, in order to reveal the biopharmaceutical aspects of the recommended administration strategies. Current administration practices were also compared with the relevant regulatory guidelines in order to assess possible differences and clinical consequences. Finally, a statistical model was developed in order to understand the choice of vehicle recommended based on the characteristics of the drug/formulation.

## METHODS

In this study, a focused search was performed on the vehicles that are globally used for mixing with dosage forms for paediatric administration. When gathering data, it was identified that the formularies previously consulted (British National Formulary for Children [BNF-C] [22] and Hospital Formulary [23]) do not differ from the formularies used in several other countries such as Australia, New Zealand, and the Netherlands. The Lexicomp Neonatal and Paediatric Dosage Handbook [24] (referred to as *Lexicomp Handbook* in this paper) was identified as a second source of information. In clinic, it is a valuable point-of-care dosing resource, designed to support medical professionals managing paediatric and neonatal patients. For the purpose of this study, the drug monographs included in this handbook were screened, with emphasis on the ‘mode of administration’ section. Drugs were included in the analysis if co-administration of the dosage forms with food, drinks or meals was suggested. Drugs for which recommendations were to take the formulations ‘with or without food/meals’ or ‘without regards to food/meals’ were also included. As this review focuses on a specific type of medicine manipulation (i.e. mixing the drug with vehicles), drugs for which only manipulation techniques were referred and/or drugs for which only water was noted as an administration vehicle were not taken into account. The information gathered from this new source was combined with information previously gathered, for a global evaluation of practices and vehicle recommendations [7, 22, 23].

Logistic regression analysis was performed to further understand the biopharmaceutical basis of the choice of recommended vehicle for medicine co-administration, using XLSTAT^®^ software (an Add-In for Excel, Microsoft^®^). This method is used to understand the effect of a series of variables on an unordered qualitative response variable (a variable which can take at least two values) [25]. The statistical analysis was performed to predict the effect of drug and formulation characteristics (namely, drug logP, drug aqueous solubility and formulation type) on the choice of vehicle type (response variable; drinks or soft foods) recommended to be mixed with paediatric medicines. The explanatory variables used were: high/low drug solubility (presented as *HighSol* and *LowSol*, respectively, accordingly to the FDA drug solubility criteria), formulation type (*Solid/Liquid*), and drug logP (presented as *Hydrophilic* for logP < 3 and *Lipophilic* if logP > 3). Prior to carrying out the statistical analysis, all the parameters used were tested for multicollinearity, to evaluate whether any of the dependent variables were highly correlated with each other. VIF values < 3 indicated absence of multicollinearity amongst the variables chosen (data not shown). The statistical analysis was described by an equation, which was built relatively to the response variable chosen as reference category (in this case, drinks as the vehicle type recommended for drug administration). Standardised coefficients were generated for each variable in the equation, allowing for the comparison of the influence of each variable to the model built. The higher the absolute value of a coefficient, the more important the weight of the corresponding variable. The obtained equation was a model of the probability associated to the type of recommended vehicle being drinks, depending on the values of the explanatory variables [25]. If the estimated probability of the event occurring is greater than or equal to 0.5, the event is classified as occurring. If the probability is less than 0.5, the event is classified as not occurring (in this case, the vehicle type recommended is not drinks, but soft foods). To build and validate the analysis, a total of 430 drug-formulation-vehicle combinations were considered; these were divided into two groups: 300 combinations were used for the construction of the model, and 130 (corresponding to ~ 30% of the total number of combinations, and randomly selected by the software) for the validation of the model. Model validation was performed by comparing the vehicle recommended in the formularies of the validation subset and the vehicle predicted by the model equation.

## RESULTS

### Mixing Medicines with Food/Drinks in the Context of Their Physicochemical Properties

The Lexicomp Handbook lists recommendations for the administration of paediatric medicines, providing information on which food or drinks to use for medicine co-administration, when applicable [24]. Supplementary Table I lists the 407 drugs (out of 1054) included in this handbook that are recommended to be mixed with food and drinks prior to oral administration, in addition to the recommended vehicles for administration. The BCS class of the drug, aqueous drug solubility and drug ionisation characteristics were added to the information collected from the Lexicomp Handbook. Eight formulation types were identified: *tablets*, *capsules*, *ampoules*, *granules*, *powder*, *solutions*, *syrup* and *suspensions*. The types of vehicles recommended were categorised into *Soft foods* (e.g. yoghurt, applesauce, fruit puree), *Drinks* (e.g. milk, juices, formula) and *Others* (e.g. meals, food, suspending agents/syrups). Recommendations for administration with water were only noted when it was an alternative to other drinks. Specific recommendations included in the drug monographs were noted, such as unsuitable vehicles, further examples of suitable vehicles for mixing, and/or the acceptable amount of vehicle to administer. Drugs for which simple manipulation techniques were given without specific suggestions for mixing with vehicles (e.g. tablets ‘may be crushed or dissolved’) were not included. Drugs for which recommendations were to take the formulations ‘with or without food/meals’ or ‘without regards to food/meals’ were included; for simplification, these recommendations will be denoted as ‘with or without food’ in this review. It is worth noting that improving palatability/taste was indicated in 2 cases, lopinavir/ritonavir tablets and ritonavir liquid, as a reason for co-administration with a vehicle. However, this information was not revealed for the remainder of the drugs. Similarly, decreasing gastrointestinal (GI) distress was indicated in 23% (94/407) of the cases as a reason for medicine co-administration with food/drinks.

The drugs previously collected from other sources [22, 23] were added to the database for further analysis, in order to obtain a global understanding of the vehicle recommendations. The database used for analysis encompassed 428 drugs (with and without paediatric indication, as long as included in the formularies reviewed), of which 77% (331/428) were included only in the Lexicomp Handbook, 5% (21/428) only in the UK formularies and 18% (76/428) in sources from both settings, although sometimes with different recommendations.

**Fig. 1:**
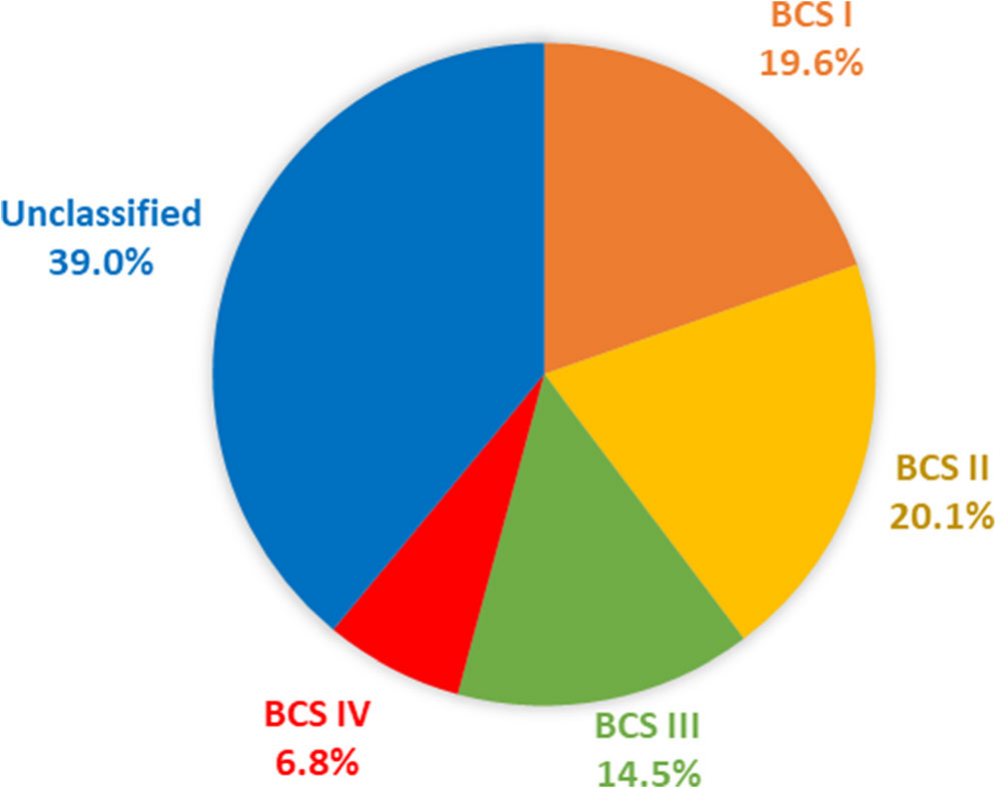
BCS classification of the drugs recommended to be mixed with food and drinks

The BCS is a regulatory framework for oral drug products for adults, which categorises drugs based on their solubility and permeability [26]. Sixty-one percent of the 428 drugs gathered were classified into one of the four BCS classes, based on information (published studies or predictive values) regarding the solubility and permeability of the drugs (Fig. 1). It was shown that most drugs suggested to be co-administered with food and drinks are drugs with high permeability (19.6% and 20.1% belong to BCS class I and II, respectively), whereas only 14.5% of the drugs belonged to BCS class III and 6.8% to BCS class IV. It should be noted that unclassified drugs (in terms of BCS class) were not considered for further analysis.

Mixing a paediatric medicine with food and drinks has been shown to affect its biopharmaceutical characteristics [7]. To further investigate this, analyses were carried out to reveal potential correlations between the BCS class of the drugs, the type of formulation administered, and the type of vehicles recommended for mixing with the drug.

#### BCS Class of the Drug *vs* Formulation Type

**Fig. 2:**
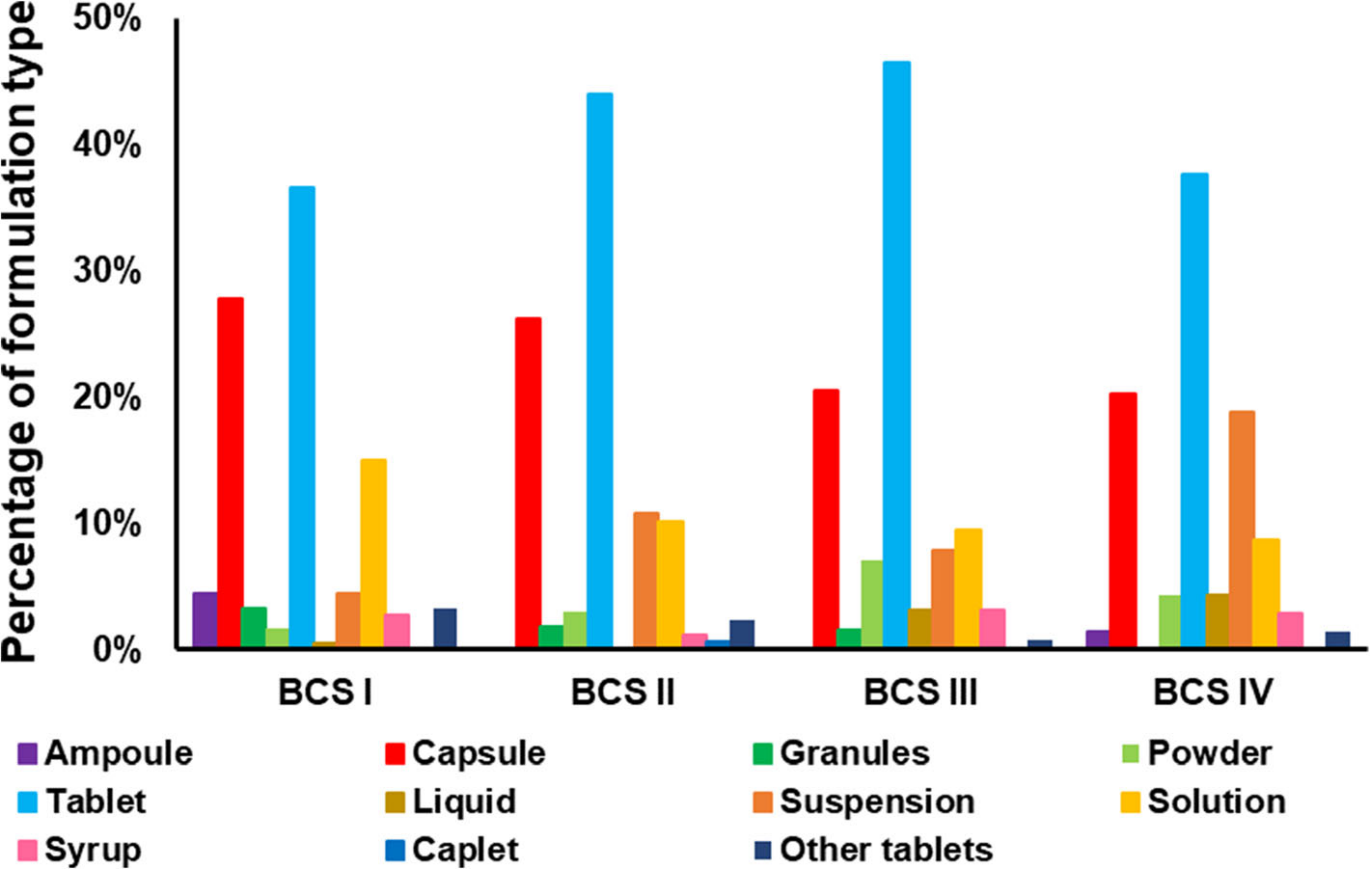
Percentage of type of formulation in relation to the BCS class of the drug

The relationship between drug BCS class and the type of formulation co-administered with food or drinks is shown in Fig. 2. Tablets and capsules were shown to be the predominant dosage forms mixed with foods or drinks, for drugs of the four BCS classes. BCS class I products formulated as solutions and BCS class IV products formulated as suspensions are also commonly recommended to be mixed with foods or drinks.

#### BCS Class of the Drug *vs* Type of Vehicle

**Fig. 3:**
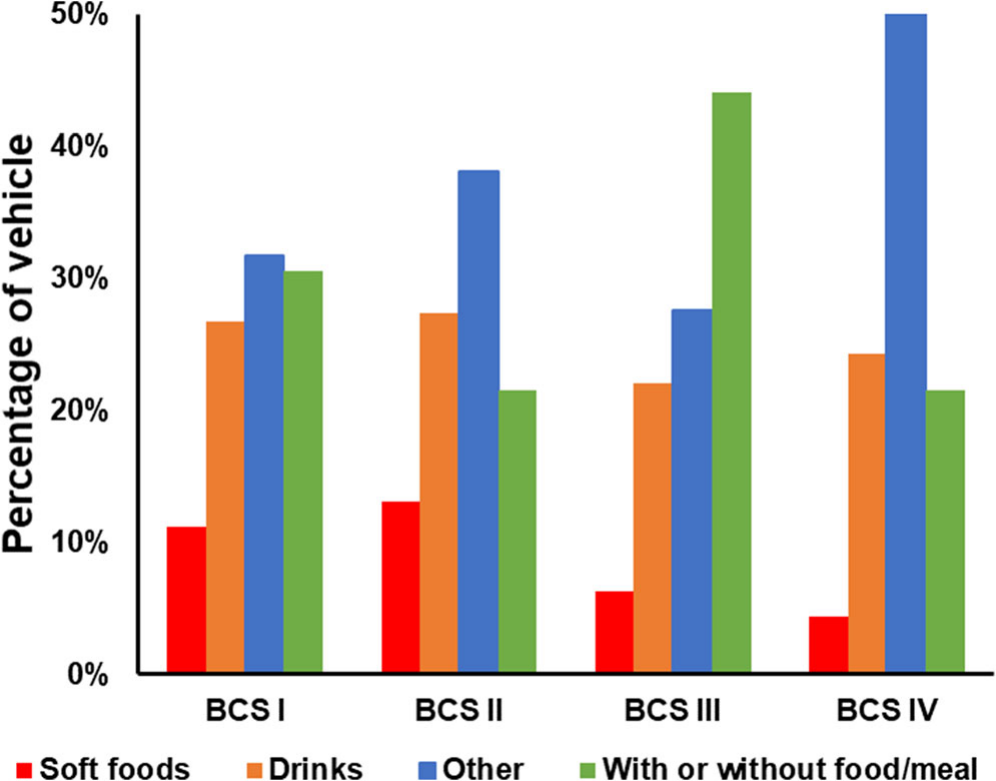
Percentage of the type of vehicle in relation to the BCS class of the drug

Figure 3 shows the prevalence of the type of vehicle used for drugs belonging to each BCS class. Vehicles of all types are recommended for mixing with all BCS classes. Soft foods are the least commonly suggested to be mixed with paediatric medicines, particularly with BCS class III and IV drugs. Meals/foods and syrups (classified as *others*) are the most commonly recommended vehicles for co-administration with drugs belonging to BCS class I, II and IV. Despite this, recommendations are often not clear on the mixing process, the type and the amount of food/meal to use. For BCS class III drugs, the most commonly suggested recommendation is to mix ‘with or without foods/meals’, which is an unclear recommendation regarding whether it is possible to mix the drug with vehicles. Drinks are commonly suggested to be mixed with paediatric medicines for drugs of all BCS classes.

Recent studies have assessed the physicochemical properties of vehicles commonly reported to be mixed with paediatric medicines for co-administration [27, 28]. Distinguished differences between the physicochemical properties (e.g. pH, surface tension, osmolality, viscosity, buffer capacity) and macronutrient composition of different food and drinks were observed, both amongst vehicles of different types (drinks *vs* soft foods) and within vehicles of the same subtype (e.g. different formulas). These differences between vehicle properties affect drug solubility and dissolution properties, particularly of poorly soluble drugs [29–31]. For example, solubility studies of mesalazine and montelukast performed in drinks and soft foods resulted in considerably different drug solubility values in each vehicle, being significantly affected by different vehicle physicochemical properties and macronutrient composition [29–31]. This vehicle-dependent impact on drug properties could compromise drug bioavailability and should be taken into consideration during paediatric product development.

#### Type of Vehicle Recommended *vs* Type of Formulation

**Fig. 4:**
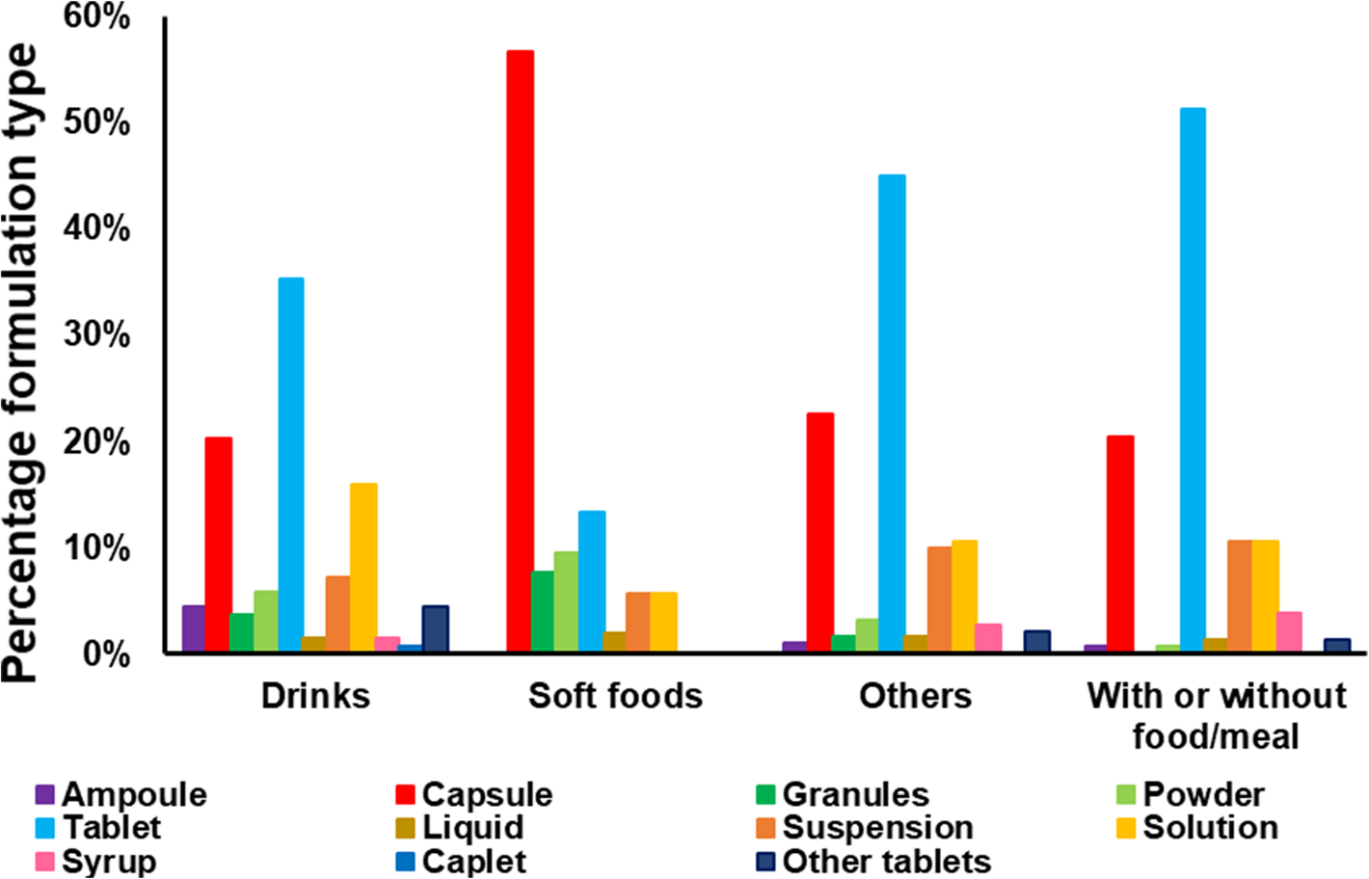
Percentage of the type of vehicle recommended in relation to the type of formulation

The relationship between the type of vehicle recommended for medicine co-administration and the type of formulation is presented in Fig. 4. Ampoules for IV administration are mainly recommended to be mixed/diluted with drinks and administered orally. In some cases, such as for topotecan ampoules, the recommendation is to mix with acidic drinks (e.g. apple juice); however, this type of recommendation should not be generalised since depending on the drug this practice might affect drug stability. Soft foods are mainly suggested for mixing with capsule formulations. All vehicle types are reported for mixing with liquids, solutions and suspensions. Apart from soft foods, all vehicle types are recommended to be mixed with syrups. Tablets are recommended to be mixed with all vehicle types, with a high prevalence of mixing with meals/food and with suspending agents/syrups for extemporaneous preparations. Mixing ‘with or without foods/meals’ is reported for all formulation types, except granules and caplets. It should be noted that for several cases, recommendations were made to mix the suggested vehicles with oral dosage forms, and so all the oral drug formulations listed in the Lexicomp Handbook as available were considered. This suggests that recommendations were possibly made based on physicochemical properties and characteristics of the drug, and not formulation.

### Effect of Drug/Formulation Properties on the Choice of the Recommended Vehicle

Although there have been many reports on the use of food and drink vehicles to facilitate administration of paediatric medicines, there are still major gaps in the knowledge of the scientific rationale for choosing which vehicle is appropriate [7, 15].

Logistic regression analysis was performed to investigate the relationship between drug/formulation variables and the type of vehicle recommended (drinks, soft foods). The statistical model is described by the following equation (Eq. 1):

1 $$ {\mathit{\Pr}}_{\left(\mathrm{drinks}\right)}=1/\left[1+{e}^{-\left(811.400+0.113\times \mathrm{LowSol}+0.460\times \mathrm{Solid}-0.143\times \mathrm{Lipophilic}\right)}\right] $$

where Pr_(drinks)_ is the probability of the vehicle type recommended to be drinks; LowSol, Solid and Lipophilic get a value of 1 or 0 depending on whether the drug/formulation has these characteristics or not, respectively. For example, for a lipophilic drug (Lipophilic = 1), with high solubility (LowSol = 0) and formulated as a tablet (Solid = 1), the probability of the vehicle type recommended to be drinks is 0.59 (meaning that drinks would be the vehicle type predicted).

**Fig. 5:**
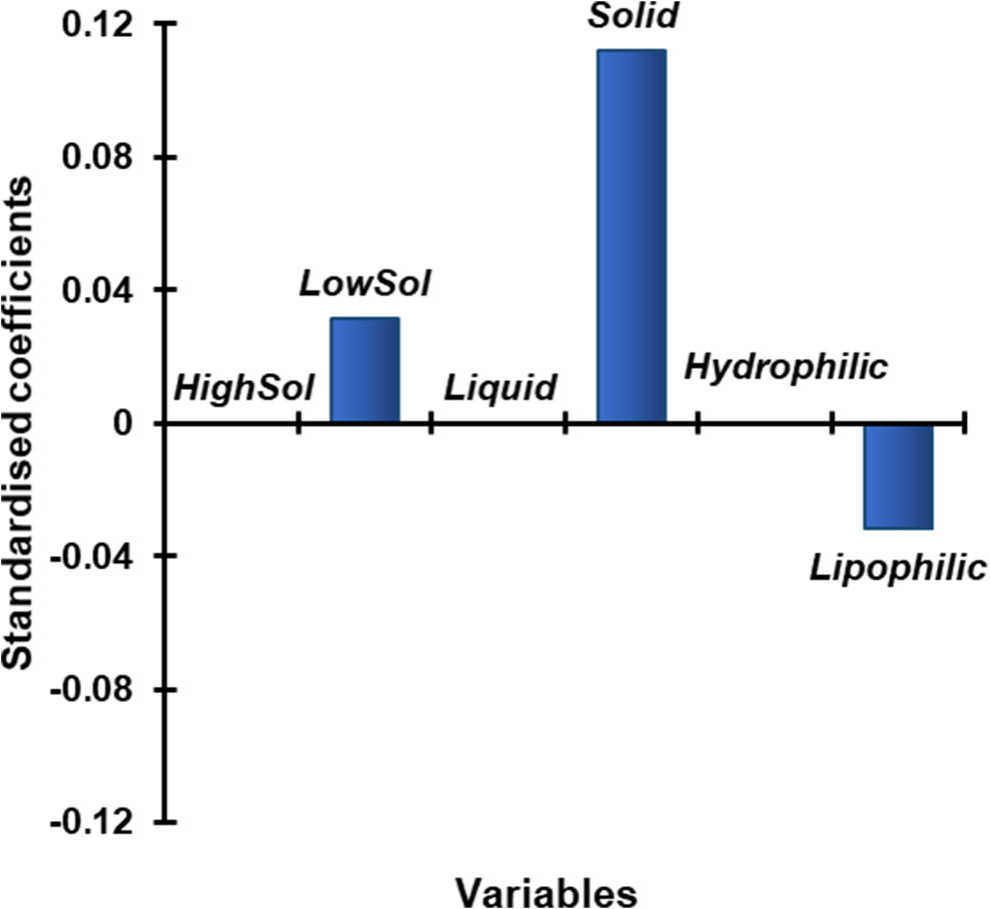
Standardised coefficients corresponding to the variables studied for the logistic regression model constructed

The standardised coefficients of each studied variable are presented in Fig. 5 and reveal that formulation type is the variable with most impact on the choice of vehicle type recommended (*p* < 0.05). In view of the model equation variables, it can be anticipated that the likelihood of drinks being the chosen vehicle type for medicine co-administration is increased if the drug is lipophilic, with low aqueous solubility and formulated in a solid form.

Model validation was performed by comparing the type of vehicle recommended in the formularies and the vehicle type predicted by the model equation, using 130 drug-formulation-vehicle combinations. In 60% of the cases, the logistic regression model could predict the vehicle type recommended from the tested sources, according to drug and formulation characteristics.

Overall, this analysis was a first approach towards defining a correlation/rationale between the type of vehicle suggested for mixing and the drug and formulation properties. The developed model provides an insight on which vehicle type is recommended for use with basis on the biopharmaceutical properties of the drugs/formulations. It has a reasonably good predictive ability, with predicted and calculated vehicle recommendations in the test set showing good agreement. Nevertheless, given that the model is currently based on a dataset comprising a limited number of sources, further work is required to verify and extend the approach. Despite its limitations, the analysis described provides information to generate awareness and discussion towards co-administration practices of paediatric medicines, within the clinical and scientific communities. In the future, it would be useful to include information from other formularies not identified in this review to further refine and validate the model constructed.

## Discussion

### Discrepancies in Recommendations Reported—a Global Perspective

In this review, the availability of drug products recommended to be mixed with food and drinks was assessed using two datasets: (i) the list of drugs gathered after consulting the Lexicomp Handbook [24], and (ii) the database previously collected from two sources (BNF-C [22] and a Hospital Formulary [23]). Over half of the drugs for which mixing with a vehicle was suggested in the Lexicomp Handbook were not included in the UK formularies. Although it is not completely clear how the recommendations were established, a possible explanation for this is the discrepancy observed in the number of drugs included in the sources (e.g. the Lexicomp Handbook included 1054 drug monographs whereas the UK formularies included less than half that number). In addition, 47% of the drugs were included in both datasets, but with no vehicle suggestions for medicine co-administration in the UK. For example, terbinafine is recommended to be mixed with non-acidic foods in the Lexicomp Handbook, but not in the UK formularies even though it was included in the formularies consulted. A more concerning issue arises in the cases of tenofovir disoproxil fumarate, sodium phenylbutyrate and risperidone (Table I). In the first case, the BNF-C and Lexicomp Handbook warn against mixing with liquids, whereas mixing of the granules with orange juice or water is advised in the Hospital Formulary. In the case of sodium phenylbutyrate, the Lexicomp Handbook advises against mixing with acidic drinks whereas fruit juices are recommended in the UK formularies. Similarly, risperidone formulations are suggested to be mixed with coffee in the formularies, whereas this drink is advised against mixing with the drug in the Lexicomp Handbook.

**Table I:** Differences in Recommendations Between the Different Sources Consulted

Drug	Sources	
	Lexicomp Handbook (24)	BNF-C (22) and Hospital Formulary (23)
Risperidone	Mix with water, orange juice, or low-fat milk Do not mix with coffee or tea	Mix with milk, juice, coffee, tea, fruit juice, orange juice [23]; Mix with non-alcoholic drinks except tea [22]
Sodium phenylbutyrate	Avoid mixing with acidic beverages e.g. most fruit juices or colas, food, meal or feeding	Mix with fruit juice [22], meals, milk [23]
Tenofovir disoproxil fumarate	Do not mix with liquids Mix with 2–4 oz of applesauce, baby food, yoghurt	Mix with soft foods e.g. yoghurt, applesauce [22]; Mix with orange juice [23]

### Medicine Co-administration with Food and Drinks—from Regulatory Guidance to Reported Recommendations and Practices

The widespread use of *off-label* and unlicensed medicines for the paediatric population confirms that the currently available commercial products do not meet the needs of this population. Medicines are often manipulated prior to administration due to unacceptability of the dosage form to the patient or unavailability of the needed dose. Medicine co-administration with vehicles is the most practiced manipulation strategy in paediatrics; however, no recommended testing methodology or uniform criteria to define what is classed as globally acceptable for the different paediatric age groups (e.g. in terms of flavour, texture and composition) have been set to predict the possible impact of medicine co-administration with vehicles on drug product performance [6, 11, 32].

Current guidance has begun addressing the recommended strategies for paediatric medicine development, acceptability and administration, including drug manipulation techniques and with special emphasis on co-administration of medicines with food and drinks [5, 8, 15, 33]. The most recent example is the Food and Drug Administration (FDA) draft guidance released in 2018, in which vehicle selection approaches and *in vitro* testing for co-administration of paediatric medicines are recommended [15]. The three main purposes of this draft guidance are: (i) to give recommendations on vehicle selection, (ii) to describe standardised *in vitro* methods for evaluating vehicle compatibility, and (iii) to provide suggestions on product labelling for communication of acceptability (or unacceptability) of vehicles intended for mixing with the medicine.

In the following subsections, the considerations provided in current regulatory guidance regarding vehicle selection and testing will be discussed and compared with reported recommendations, gathered from the sources consulted, and reported healthcare practices [5, 15, 33].

#### Vehicle Selection: *In Vitro* Assessment of Drug Product-Vehicle Compatibility and Use in Practice

Regulatory guidance states that *in vitro* compatibility studies should be performed when co-administration of medicines with food or drinks is intended. It is recommended that comprehensive suitability determinations are conducted to evaluate the potential impact of the proposed vehicle on drug behaviour and provide guidance on the appropriate vehicle to use in the target age group. These assessments include: (i) potency assays, to quantify the amount of drug in the drug product-vehicle mixture, evaluate drug product performance and support the recommended use time of the mixture after preparation; (ii) integrity testing, to verify if the drug substance quality attributes are maintained after mixing with a vehicle; (iii) stability assessments, to support instructions for the mixture preparation and labelled use time of the mixture; (iv) dose uniformity/homogeneity testing; and (v) drug release/dissolution testing, to determine possible changes in drug behaviour.

Ideally, food and drinks which have been proven to cause no appreciable effect on medicine performance should be proposed as vehicles. It is advised that drug product information (labelling, SmPC, PIL) should also include instructions on vehicles found unacceptable, including the rationale for avoiding their use as vehicles for medicine co-administration [15]. For example, a soft food like applesauce should be deemed inappropriate if the targeted patient population are infants still consuming a liquid diet, even if the mixture vehicle-drug product is physicochemically stable [15].

In practice, according to the administration techniques reported by healthcare professionals, carers and parents, it is common to mix formulations with foods and drinks that have not been evaluated (i.e. not mentioned in the SmPC, PIL or product labelling) [13, 14, 34]. Consequently, an unsuitable vehicle might be used, which may lead to possible changes in drug performance *in vivo*. This might be critical since different food and drinks can have dissimilar effects on a paediatric medicine due to their physicochemical properties and might significantly impact drug bioavailability and, consequently, therapeutic efficacy [9, 35]. For example, crushing of gastro resistant dosage forms, such as NSAID drugs, to mix with a vehicle can alter drug absorption and efficacy and/or cause irritation of the GI mucosa and, ultimately, may increase the risk of side effects, such as formation of GI ulcers. Stability and compatibility studies of tegaserod from crushed tablets in soft food and drinks (water, apple juice, orange juice, and applesauce) revealed that whilst the drug was stable in and compatible with these vehicles, the dissolution profiles of the crushed tablets in orange juice and applesauce were not comparable with those of intact tablets [36].

The FDA draft guidance provides a list of 27 vehicles commonly used for medicine co-administration (reproduced in Table II), which includes the most predominant vehicles used in both inpatient and outpatient settings, such as drinks (e.g. fruit juices), yoghurts and banana purée [14, 34]. In the formularies consulted [22–24], a predominant vehicle type is not recommended, probably due to the lack of rationale behind vehicle selection. When comparing the information gathered from the consulted formularies/handbook with reports from healthcare professionals and the FDA draft guidance, several discrepancies were found in recommendations [7, 15, 24]. For example, only 44% (12/27) of the vehicles listed in the FDA draft guidance were referenced more than 5 times in the sources consulted, 15% (4/27) of the vehicles are referenced between 1 and 3 times in the sources consulted, and 41% (11/27) are not specifically mentioned as recommendation vehicles. Banana purée is one of the vehicles included as being frequently used in practice (both according to reports from healthcare professionals and the FDA guidance) but is not clearly stated as an example in any of the sources consulted [14, 22–24]. Concerning issues may arise from these differences; for instance, juices are frequently used vehicles in practice but, in the formularies consulted, using fruit juices for medicine co-administration is advised against in the cases of several drugs (e.g. bosentan tablets, ethambutol tablets and etravirine tablets) (supplementary Table I) [7]. Moreover, although vehicles with higher viscosity are frequently used (e.g. banana purée, yoghurt), vehicle viscosity has been shown to negatively affect the dissolution of different drugs [30, 31, 37].

**Table II:** Commonly Used Soft Foods and Drinks (*Reproduced from* [15])

Soft foods	Drinks
Apples (purée)	Apple juice
Applesauce	Buttermilk
Baby food (unstrained)	Coconut milk
Bananas (puree)	Cranberry juice
Carrots (puree)	Water
Chocolate pudding	Grapefruit juice
Fruit jellies	Infant formula
Fruit jam	Milk
Honey	Orange juice
Maple syrup	Pineapple juice
Orange marmalade	Soybean milk
Peanut butter	
Rice pudding	
Strawberries (puree)	
Strawberry jam	
Yoghurt	

Overall, in practice there seems to be no clear rationale behind vehicle selection for use in medicine co-administration. For most drugs, information of possible co-administration with vehicles is not included in the product information (labelling, SmPC nor PIL); therefore, the possible impact of this practice on drug performance is often unaddressed [6, 7, 11, 38]. Recognising this, the FDA draft guidance establishes a clear rationale on the most correct approach for vehicle selection and standardised age-appropriate testing methodologies. Vehicle selection and age-appropriate compatibility methodologies of drug-formulation-vehicle should be addressed during paediatric product development, to understand the vehicle impact on the drug product and the implications of medicine co-administration on drug clinical outcomes. In this context, a decision tree for vehicle selection is available on the FDA draft guidance, presented as a recommendation and not a mandatory requirement during paediatric drug development [15]. A complicating factor for the establishment of uniform practices is the incorrect assessment of the acceptability of the product-vehicle mixture, in terms of flavour, texture, mouthfeel, and age-related responses to physical characteristics of the mixture [38]. For example, pharmacokinetic studies have been performed with applesauce, which is not always well accepted amongst the paediatric population (e.g. in younger age groups whose diet consists mostly of liquids) [27]. Therefore, the potential acceptance of the paediatric population and vehicle uniform composition in different countries should be a focus point in the recommendations. Ultimately, it is necessary to fully establish and regulate assessment criteria and perform appropriate studies to provide better guidance for healthcare practitioners, patients and carers regarding medicine co-administration with vehicles in the paediatric population.

#### Volume of Vehicle

The suggested volume of vehicle to use for mixing with solid oral dosage forms should take into consideration the age, size, and average consumption of the vehicle by the targeted patient population. For example, children younger than 2 years old may not be able or willing to ingest large volumes of drinks or soft foods at one time. Regulatory guidance from both the FDA and the European Medicines Agency (EMA) states that the typical volume of vehicle administered to a paediatric patient should be swallowable in one unit to ensure administration of the complete drug dose, whilst facilitating swallowing and providing acceptable taste-masking [15, 33]. Volumes between 5 and 15 mL have been proposed as acceptable and are normally preferable, which means that exploring alternative vehicles should be considered if a large volume is required [38]. However, in adult studies recently conducted to investigate the administration of paediatric formulations mixed with vehicles, the volume of vehicles used varied between one tablespoon and 120 mL [6]. Moreover, when looking at the recommendations gathered (supplementary Table I), it is observed that very different volumes of vehicles (ranging from 5 to 200 mL) are suggested to be mixed with the different drugs, although no justification is provided for the suggestions. For example, imatinib tablets 100 and 400 mg can be mixed with 50 and 200 mL of water or apple juice, respectively; lansoprazole capsules can be opened and mixed with 60 mL of juice or 1 tablespoon of soft foods; topotecan capsules can be opened and mixed with 30 mL of juice; and pantoprazole suspension can be mixed with 5 mL of juice.

The use of different volumes of vehicles can be prejudicial for the clinical outcome. For example, using a large amount of vehicle (e.g. one pot of yoghurt) might lead to decreased accuracy in dose delivery, especially if the whole product is not consumed; conversely, use of a very small volume of vehicle (e.g. less than 5 mL) might not properly improve the palatability of the medicine and result in the patient refusal to consume it. Thus, further studies should be conducted towards defining age-appropriate volumes to consume and a mandatory regulatory statement concerning the appropriate volumes for product testing should be provided to ensure a more unified approach.

#### Mixture Preparation and Handling

Standardisation of the preparation and use instructions for the drug product-vehicle mixture is important, as ambiguity in instructions or incomplete information can lead to unintended outcomes, including decreased accuracy in dose delivery and/or misuse of the drug product. For details on drug manipulation techniques and potential implications, the reader is referred to our previous review in which these topics are discussed extensively [7]. Therefore, the FDA draft guidance states that the complexity of the preparation, homogeneity of the mixture, and handling procedures should be considered by the manufacturer [15]. One idea that has been proposed to facilitate administration, whilst ensuring dosing accuracy, is to include an oral syringe or measuring spoon with the drug product along with clear use instructions to avoid administration errors [15].

In practice, no standardised rationale seems to be used for administration practices of medicines to paediatrics. Drug manipulation practices as reported by parents, carers and healthcare professionals in inpatient and outpatient settings have been recently evaluated [13, 34, 39]. For example, in a study recently conducted in the Netherlands, it was revealed that only 55% of medicines were manipulated according to the instructions or recommendations of the SmPC or PIL [34]. The main reasons for drug manipulation were found to be dose adjustment, taste improvement or feeding tube administration, with 52.3% of the nurses interviewed admitting to having deviated from hospital protocols for manipulation [34]. Similarly, manipulation of oral dosage forms has been shown to be common practice amongst parents, carers and healthcare professionals in other paediatric hospitals of different countries (e.g. UK, Australia) [13, 14, 39].

In general, the predominant reasons for manipulation have been shown to differ between the inpatient and outpatient settings. Manipulation by parents and carers is usually performed for taste and dose adjustment, whilst healthcare professionals most often use manipulation for administration through a feeding tube, or for dose reduction [34, 40, 41]. This difference probably results from: (i) the more extensive formularies of inpatient pharmacies, which allow a more precise dosing with compounded dosage forms of different strengths, clinically supported by vehicle recommendations, and (ii) the higher prevalence of feeding tubes in the inpatient setting [34, 40]. Regardless of the setting, the method used for mixture preparation and handling can differ depending on the person performing it, which can lead to dose accuracy inconsistencies [7]. The risk of errors related to the drug manipulation will also increase if incorrect information is transferred from the healthcare professional to the parent and carer.

Overall, differences are still observed between current guidance recommendations and reported administration practices. This highlights the need for additional in-service training of the healthcare professionals and, consequently, of parents and carers regarding drug manipulation, in order to fully harmonise medicine co-administration practices and avoid potential issues in drug product performance.

#### Time Between Preparation and Administration of the Mixture

The FDA draft guidance states that the drug product-vehicle mixture should exhibit no change in potency (as determined by a validated assay) nor in drug release characteristics over the time period proposed in the product information [15]. It is generally recommended that prepared drug product-vehicle mixtures should be administered immediately or as directed in the product information, in order to avoid potential dosing errors and/or microbiological contamination of the mixture [15]. The proposed timeframe for administration of the mixture should be supported by product quality assessments in which the physicochemical stability of the mixture is ensured. If the mixture is intended to be used more than 2 h after preparation, microbiological testing should be also carried out [15].

In practice, information regarding the time frame for use of the mixture is often not indicated. Analysis of the recommendations gathered in the sources consulted, as well as of recent reports on common practices in healthcare settings, revealed that information regarding the importance of immediate administration after mixture preparation is not provided for most of the drugs suggested to be mixed with vehicles [7, 24]. For example, this information was only available for 2 of the 408 drugs collected in the Lexicomp Handbook (supplementary Table I) [24]. These were: amoxicillin tablets (mixture should be administered ‘immediately’), and ivacaftor granules (mixture should be consumed ‘within 1 h’).

The time between the preparation and administration of the mixture may influence drug stability, solubility and dissolution and, subsequently, its oral absorption. In recent studies, we have assessed the effect of delaying the testing of drug product-vehicle mixtures (by 4 h after their preparation) on the stability and dissolution of two poorly soluble compounds (mesalazine and montelukast) and their formulations [30, 31, 42]. It was revealed that drug loss could occur to a small extent (< 15%) in a time-dependent manner and, consequently, concluded that administration of the mixtures should ideally be performed immediately after preparation, or at least within 4 h of preparation. An immediate administration of the mixtures would not only avoid potential drug/formulation stability issues and increased risk of drug precipitation, but also prevent other vehicle-effects on drug dissolution (e.g. increased solubilisation and wetting of the formulation). Other potential consequences are the increase of risk of adverse side effects, depending on the pharmacological category of the drug [37, 43].

Overall, when mixing with a vehicle is intended, information on the time for administration of the mixture should be provided to ensure proper administration of the manipulated dosage form, whilst guaranteeing drug safety and efficacy. The establishment of unified, global practices would be helpful in avoiding possible, significant clinical outcomes.

#### Information Required for Clinical Practices of Co-administration with Food and Drinks

PILs should provide enough information to ensure that healthcare providers, patients, parents and/or carers have the essential knowledge required for appropriate use of the recommended vehicles. In regulatory guidance, a list of recommended information to include in the product information is given, and includes: (i) recommended vehicle type; (ii) detailed information on the vehicle to use, including volume and temperature; (iii) recommended critical manipulations (e.g. opening a capsule and emptying its contents or crushing a tablet); (iv) information on vehicle compatibility and mixture administration (including a succinct summary of compatibility/suitability data); and (v) a rationale for avoiding certain vehicles [5, 15, 33].

In reality, this information is scarce for most drugs, hindering the informed administration of acceptable vehicle-medicine mixtures to paediatric patients [7, 34, 38]. In addition, even when food-drug interactions are known to the healthcare professional, it is not always possible to administer the drug with acceptable vehicles due to limitations on which vehicles can be used for administration through enteral feedings [40, 44].

#### Clinical Evaluation of Medicine Co-administration Practices

Although regulatory bodies acknowledge the importance of conducting paediatric studies and their benefit for the patients, these are not considered necessary [4]. In the EU, an optional *in vivo* study to evaluate this practice is suggested in the EMA guideline on pharmaceutical development of paediatric medicines [33]. This can be a separate bioequivalence study in adults or, alternatively, paediatric clinical trials can be conducted with the vehicle of choice. Extrapolation of food-effects observed in adults into paediatric subpopulations is an unexplored and complex area due to physiological and anatomical differences between the two populations. This may result in different food effects in the paediatric population compared with adults [45, 46]. Paediatric clinical trials conducted for vehicle assessment are limited; for example, suitability tests were performed on the co-administration of montelukast paediatric formulations with formula and applesauce [6]. Paediatric clinical studies are generally conducted to investigate PK and do not always reflect paediatric administration practices, and consequently, the clinical impact of the administration of paediatric medicines with food and drinks is often not evaluated [47].

In the USA, the practice of mixing medicines with foods is described in the FDA guidance on Food-Effect Bioavailability and Fed Bioequivalence Studies; studies in healthy adult volunteers are usually requested and accepted and, additionally, *in vitro* and *in silico* tests can be accepted as supportive evidence [11, 47, 48]. In this context, a recent study described how *in vivo*, *in vitro* and *in silico* investigations were adjusted to existing knowledge available for two model drugs (one poorly and one highly soluble) [11]. Drug stability when mixed with different vehicles was confirmed and suitable vehicles for co-administration were selected, following a combination of *in vitro* dissolution and drug solubility studies and *in silico* modelling [11].

Overall, investigation of vehicle suitability as part of paediatric clinical trials would provide the highest reliability in terms of product safety and efficacy. However, introduction of additional drug administration conditions and patient recruitment difficulties might further complicate the design, execution, interpretation of results, and ultimately the outcome of clinical studies. The use of *in vitro* and/or *in silico* age-appropriate predictive tools to aid understanding of formulation performance in paediatrics would be beneficial to understand the impact of medicine co-administration with vehicles and age-related factors on drug behaviour. Furthermore, these tools could be used to predict *in vivo* clinical outcomes. Ultimately, the development and establishment of *in vitro* and/or *in silico* testing during paediatric drug development could help reduce the number of *in vivo* studies required for paediatric formulation development, and tackle ethical issues related to clinical research in the paediatric population [2].

## Conclusions

In view of the prevalence of the practice of medicine co-administration with food and drinks in paediatrics, and of the challenges still faced in the development of age-appropriate medicines, efforts should be made to reconcile the information available and provide clear, easily accessible information on vehicle suitability. Regulatory guidance indicates that clear instructions should be included on which vehicles have been demonstrated to be suitable for mixing with medicines. However, this study has shown that information on the appropriate vehicle to use is still not available for many medicines, and no clear rationale seems to guide vehicle recommendations. The absence of standard methods and criteria defining which vehicles are widely acceptable and available for the paediatric age groups, and of standard protocols for administration, complicate the compatibility studies needed to provide informed recommendations regarding this administration practice. Moreover, the absence of mandatory status leads to differences between practice and recommendations, further hindering the establishment of uniform, acceptable administration techniques. A statistical model was developed to provide an understanding on which vehicle is recommended for use in medicine co-administration practices, with the information gathered from available paediatric formularies, based on the physicochemical and biopharmaceutical properties of the drug/formulation. This could serve as a starting point towards the development of unified guidelines, where selection of a vehicle can be made based on drug/formulation characteristics. In the future, this could also be combined with information regarding the physicochemical properties and composition of the vehicles. It is evident that further information is required for the elucidation of current practices on a global perspective.

Overall, the use of *in vitro* and/or *in silico* biopharmaceutical techniques to predict *in vivo* performance in the paediatric population is warranted to further understand the propensity for possible clinical outcomes associated with this practice of medicine administration. The developmental changes in the mechanisms driving drug dissolution and absorption are yet to be fully understood, which further complicates paediatric formulation development. Despite these gaps, the understanding and development of age-appropriate formulations is increasing, with physiologically based pharmacokinetic (PBPK) software platforms being increasingly used to predict drug disposition in paediatrics. These platforms might be used to predict the impact of drug manipulations and/or to identify the best suited vehicle for drug manipulation, addressing the need for improving information regarding the co-administration of medicines with foods and drinks.

Patients would benefit from having access to medicines known to be acceptable during both clinical evaluation and subsequent therapy, with certainty of achieving a successful clinical outcome and improved quality of life. Healthcare professionals would benefit from obtaining complete training on this practice in order to be informed on possible clinical outcomes and correctly train parents and carers. Thus, efforts should be made to reconcile the information available and provide parents, carers and healthcare professionals with more uniform and established, scientifically-based regulation.

## Electronic supplementary material

ESM 1 (DOCX 106 kb).
