# Supplementary material for: Co-administration of Paediatric Medicines with Food and Drinks in the Context of Their Physicochemical Properties—a Global Perspective on Practices and Recommendations
Source: AAPS J. 2020 Mar 4;22(2):54. doi: 10.1208/s12248-020-0432-9 (PMC7056676; doi:10.1208/s12248-020-0432-9)
Supplement: Supplementary file 1 — (DOCX 106 kb). [file 12248_2020_432_MOESM1_ESM.docx]

**Supplementary material**

**Supplementary Table 1.** Drugs recommended to be mixed with food and drinks, according to the Neonatal and Pediatric Dosage Handbook (1).

| **Drug** | **BCS Class** | **Dosage form** | **Drug ionisation ^¥^** | **Aqueous Solubility (mg/mL)**  **(2)** | **Mixed with** | | | **Notes** |
| --- | --- | --- | --- | --- | --- | --- | --- | --- |
|  |  |  |  |  | **Drinks** | **Soft-foods** | **Others** |  |
| **Abacavir** | III (3) | Solution, Tablet | Weak base | 1.21 |  |  | X food |  |
| **Abacavir and Lamivudine** | - | Tablet | - | - |  |  | X food |  |
| **Abacavir, Dolutegravir and Lamivudine** | - | Tablet | - | - |  |  | X food |  |
| **Abacavir, Lamivudine and Zidovudine** | - | Tablet | - | - |  |  | X food |  |
| **Acarbose** | III (3) | Tablet | Amphoteric | 108.0 |  |  | X meal |  |
| **Acetaminophen** | I (3) | Caplet | Amphoteric | 4.15 |  |  | X food* |  |
| **Acetaminophen and codeine** | - | Capsule, Solution, Suspension, Tablet | - | - |  |  | X food* |  |
| **Acetazolamide** | IV (3) | Capsule ER, Tablet, Solution | Amphoteric | 2.79 |  |  | X food*, cherry or chocolate syrup |  |
| **Acetylcysteine** | I (4) | Solution | Weak acid | 5.09 | X cola, orange juice, or other soft drink |  |  |  |
| **Acyclovir** | III (3) | Capsule, Tablet, Suspension | Amphoteric | 2.5 |  |  | X food* |  |
| **Adefovir** | III (5) | Tablet | Amphoteric | 2.02 |  |  | X food* |  |
| **Albendazole** | II (3) | Tablet | Amphoteric | 0.0228 |  |  | X food* |  |
| **Albuterol** | I (3) | Syrup, Tablet | Amphoteric | - |  |  | X food* |  |
| **Alcohol (ethyl)** | - | Ampoule | Neutral | - | X water, juice |  |  |  |
| **Allopurinol** | I (3) | Tablet | Weak acid | 5.88 | X fluid |  | X cherry syrup |  |
| **Almotriptan** | III (6) | Tablet | Weak base | 0.121 |  |  | X food* |  |
| **Aluminium Hydroxide** | IV (3) | Capsule, Syrup | Amphoteric | - |  |  | X meal |  |
| **Amantadine** | - | Capsule, Tablet,  Syrup | Weak base | 6.29 |  |  | X food* |  |
| **Amiloride** | III (3) | Tablets | Weak base | 1.22 | X milk |  | X food |  |
| **Aminocaproic Acid** | - | Solution, Tablet,  Syrup | Amphoteric | 505 |  |  | X food* |  |
| **Amiodarone** | II (7) | Tablet | Weak base | 0.00476 |  |  | X meal | Do not administer with grapefruit juice |
| **Amitriptyline** | I (3) | Tablet | Weak base | 0.0045 |  |  | X food* |  |
| **Amlodipine** | III (3) | Tablet | Weak base | 0.0753 |  |  | X food* |  |
| **Amoxicillin** | I (8) | Tablet | Amphoteric | 0.958 | X formula, milk, juice |  |  | Administer immediately after mixing |
| **Amoxicillin and clavunate** | - | Tablet, Suspension | - | - | X formula, milk, juice |  |  |  |
| **Amphetamine** | III (3) | Tablet, Suspension | Weak base | 1.74 |  |  | X food* |  |
| **Anagrelide** | - | Capsule | Weak base | 0.279 |  |  | X food* |  |
| **Aprepitant** | IV (9) | Capsule | - | 0.0194 |  |  | X food* |  |
| **Arginine** | - | Powder | Amphoteric | - |  |  | X meal |  |
| **Aripiprazole** | IV (3) | Tablet, Solution, Suspension | Amphoteric | 0.00777 | X liquid |  |  |  |
| **Artemether and lumefantrine** | - | Tablet | **-** | **-** | X water |  | X meal | Crush tablet and mix with water, followed with food, milk, formula, pudding, porridge or broth |
| **Ascorbic acid** | III (3) | Capsule, Powder, Syrup, Tablet | Weak acid | 400 |  |  | X food |  |
| **Aspirin** | II (10) | Tablet, Caplet | Weak acid | 1.46 | X water, milk |  | X food* |  |
| **Atazanavir** | II (11) | Powder | Amphoteric | 0.00327 | X beverage^1^ | X ^2^ | X food | ^1^ *e.g.* milk, water, formula  ^2^*e.g.* yoghurt or applesauce |
| **Atenolol** | III (3) | Tablet | Weak base | 13.3 |  |  | X food |  |
| **Atomoxetine** | I (3) | Capsule | Weak base | 0.0039 | X water, liquids |  | X food |  |
| **Atorvastatin** | II (3) | Tablet | Weak acid | 0.00063 |  |  | X food |  |
| **Atovaquone** | II (7) | Suspension | Weak acid | 0.000796 |  |  | X food, high fat meal |  |
| **Auranofin** | - | Capsule | - | 0.151 |  |  | X food |  |
| **Azithromycin** | II (7) | Tablet | Weak base | 0.514 |  |  | X food |  |
| **Baclofen** | - | Tablet | Amphoteric | 0.712 | X milk |  | X food |  |
| **Balsalazide** | - | Capsule, Tablet | Weak acid | 0.0621 |  | X applesauce (capsule) | X food |  |
| **Benazepril** | I (3) | Tablet | Amphoteric | 0.0022 |  |  | X food |  |
| **Benztropine** | - | Tablet | Weak base | 0.0012 |  |  | X food |  |
| **Betaine** | - | Powder | Weak base | 1.56 |  |  | X food |  |
| **Biotin** | - | Capsule, Tablet | Weak acid | 0.22 |  |  | X food |  |
| **Bosentan** | - | Tablet | Amphoteric | 0.00904 | X non-acidic liquid |  | X meal | Avoid grapefruit and grapefruit juice |
| **Brivaracetam** | - | Tablet | Amphoteric | 46.8 | X liquid |  | X food |  |
| **Bromocriptine** | - | Capsule, Tablet | Amphoteric | 0.0858 |  |  | X food* |  |
| **Budesonide** | II (4) | Capsule | Neutral | 0.0457 |  | X applesauce (capsule) | X meals |  |
| **Bumetanide** | - | Tablet | Amphoteric | 0.0257 |  |  | X food* |  |
| **Bupropion** | I (3) | Tablet | Weak base | 312 |  |  | X meal |  |
| **Buspirone** | I (3) | Tablet | Weak base | 0.588 |  |  | X food* |  |
| **Busulfan** | - | Tablet | Neutral | 69 |  |  | X meal |  |
| **Butalbital, Acetaminophen and Caffeine** | - | Tablet, Capsule | - | - |  |  | X food |  |
| **Caffeine** | I (12) | Solution, Tablet | Neutral | 21.6 |  |  | X meal |  |
| **Calcitrol** | - | Capsule | - |  |  |  | X meal |  |
| **Calcium acetate** | - | Capsule, Tablet | - | 147 | X fluids |  | X meal |  |
| **Calcium carbonate** | - | Capsule, Tablet, Suspension | - | 128 | X fluids |  | X meal |  |
| **Calcium citrate** | - | Capsule, Tablet, Suspension | - | 4.12 | X fluids |  | X food |  |
| **Calcium glubionate** | - | Syrup | - | 47.2 | X fluids |  | X meal |  |
| **Calcium gluconate** | - | Capsule, Tablet | - | 44.2 | X fluids |  | X meal |  |
| **Calcium lactate** | - | Capsule, Tablet | - | 191 | X fluids |  | X meal |  |
| **Candesartan** | II (3) | Tablet | Amphoteric | 0.00667 |  |  | X meal |  |
| **Carbamazepine** | II (3) | Chew tablet, Suspension, Tablet ER, Capsule ER | Neutral | 0.152 | X liquid medicinal agents (suspension) | X ^1^  (capsule) | X food, meal*  (tablet) | ^1^ *e.g.* applesauce |
| **Carvedilol** | II (3) | Tablet, Capsule | Amphoteric | 0.00444 |  | X applesauce ONLY (ER capsules) | X food (tablet)* |  |
| **Castor oil** | - | Oil (discontinued) | - | - | X milk, juice, carbonated beverage |  |  |  |
| **Cefaclor** | III (3) | Capsule, suspension | Amphoteric | 10 |  |  | X meal |  |
| **Cefadroxil** | III (13) | Capsule, Tablet, Suspension | Amphoteric | 1.11 |  |  | X food |  |
| **Cefdinir** | IV (3) | Capsule, Suspension | Weak acid | 0.0878 |  |  | X food* |  |
| **Cefditoren** | II (3) | Tablet | Amphoteric | 0.0441 |  |  | X meal |  |
| **Cefixime** | IV (3) | Capsule, Tablet, Suspension | Amphoteric | 0.0551 |  |  | X food |  |
| **Cefpodoxime** | IV (3) | Tablet, Suspension | Amphoteric | 0.185 |  |  | X food (tablet) |  |
| **Cefprozil** | III (3) | Table, Suspension | Amphoteric | 0.055 |  |  | X food |  |
| **Ceftibuten** | II (14) | Capsule | Amphoteric | 0.0705 |  |  | X food |  |
| **Cefuroxime** | IV (3) | Tablet, Suspension | Weak acid | 0.284 |  |  | X food |  |
| **Celecoxib** | II (3) | Capsule | Weak acid | 0.00503 |  | X applesauce | X food (doses < 200 mg) * |  |
| **Cephalexin** | IV (15) | Capsule, Tablet, Syrup | Amphoteric | 1.789 |  |  | X food |  |
| **Cetirizine** | III (3) | Capsule, Tablet,  Suspension | Amphoteric | 0.101 |  |  | X food |  |
| **Charcoal, activated** | - | Liquid, Suspension | - | 0.0 | X orange juice, |  | X chocolate syrup | Avoid adding chemicals, dairy products, syrups (Actidose) |
| **Chenodiol** | - | Tablet | Weak acid | 0.0899 |  |  | X food |  |
| **Chloral hydrate** | - | Capsule (discontinued) | Weak acid | 43.4 | X water, fruit juice, ginger ale, formula |  |  |  |
| **Chloroquine** | II (3) | Tablet | Weak base | 0.0175 |  |  | X meal, chocolate syrup* |  |
| **Chlorothiazide** | IV (16) | Tablet, Suspension | Weak acid | 0.398 |  |  | X food |  |
| **Chlorpheniramine** | I (3) | Liquid, Tablet, Suspension | Weak base | 0.0519 |  |  | X food |  |
| **Chlorpromazine** | II (3) | Solution, Tablet | Weak base | 0.00417 | X water, milk |  | X food |  |
| **Chlorthalidone** | - | Tablet | Weak acid | 0.0528 |  |  | X food |  |
| **Chlorzoxazone** | - | Tablet | Weak acid | 2.96 |  |  | X food |  |
| **Cholecalciferol** | - | Capsule, Liquid, Tablet | Neutral | 0.00038 |  |  | X food |  |
| **Choline magnesium trisalicylate** | - | Tablet, Liquid | - | - | X milk, fruit juice |  | X food* |  |
| **Cimetidine** | III (3) | Solution, Tablet | Amphoteric | 0.816 |  |  | X food |  |
| **Ciprofloxacin** | III (3) | Tablet, Suspension | Amphoteric | 1.35 |  |  | X food* | Dairy foods reduce absorption |
| **Citalopram** | I (3) | Tablet, Solution | Weak base | 0.0059 |  |  | X meal |  |
| **Clarithromycin** | II (3) | Tablet, Suspension | Weak base | 0.0003 |  |  | X meal, food |  |
| **Clemastine** | - | Tablet, Syrup | Weak base | 0.0004 |  |  | X food |  |
| **Clindamycin** | I (4) | Solution, Capsule | Amphoteric | 0.031 |  |  | X meal |  |
| **Clobazam** | - | Tablet | Neutral | 0.164 |  | X applesauce |  |  |
| **Clomipramine** | I (17) | Capsule | Weak base | 0.014 |  |  | X food, cherry syrup* |  |
| **Clonazepam** | - | Tablet | Amphoteric | 0.011 |  |  | X food |  |
| **Clonidine** | III (3) | Liquid  Tablet | Weak base | 0.48 |  |  | X meal |  |
| **Clopidogrel** | II (3) | Tablet | Weak base | 0.051 |  |  | X food |  |
| **Clorazepate** | - | Tablet | Amphoteric | 0.025 |  |  | X food |  |
| **Clozapine** | II (3) | Tablet, Suspension | Weak base | 0.012 |  |  | X food |  |
| **Codeine** | III (3) | Tablet, Solution | Amphoteric | 0.578 |  |  | X food |  |
| **Colchicine** | III (3) | Tablet, Capsule | Neutral | 45 |  |  | X meal |  |
| **Colesevelam** | - | Granules, Tablet | - | Insoluble | X liquid (tablet) |  | X meal |  |
| **Colestipol** | - | Granules, Tablet | - | Insoluble | X beverage, liquid (tablet) |  | X soups, cereals, pulpy fruits^1^ | ^1^ *e.g.* pineapple, peaches, pears |
| **Cortisone** | - | Tablet | Weak acid | 0.0278 (acetate) | X milk |  | X food, meal* |  |
| **Cyanocobalamin** | III (2) | Liquid, Tablet | - | 12.5 |  |  | X food |  |
| **Cycloserine** | - | Capsule | Amphoteric | 877 |  |  | X meal |  |
| **Cyclosporine** | II (4) | Solution | Weak acid | - | X milk, chocolate milk, orange or apple juice |  |  |  |
| **Danazol** | - | Capsule | Amphoteric | 0.0176 |  |  | X fatty meal |  |
| **Dantrolene** | - | Capsule | Weak acid | 0.0805 | X juice or liquid |  |  |  |
| **Dapsone** | IV (3) | Tablet | Weak base | 0.284 |  |  | X meal* |  |
| **Darunavir** | - | Tablet | - | 0.0668 |  |  | X food | If co-administered with ritonavir, food is required |
| **Deferasirox** | II (4) | Tablet | - | 0.0343 | X water, other liquids |  |  |  |
| **Desipramine** | - | Tablet | Weak base | 0.0396 |  |  | X food* |  |
| **Desloratadine** | I (3) | Tablet, Syrup | Weak base | 0.00395 |  |  | X food |  |
| **Dexamethasone** | III (3) | Tablet, Solution | Weak acid | 0.0505 | X milk |  | X food, syrup |  |
| **Dexchlor-pheniramine** | - | Syrup | - | 0.0519 |  |  | X food |  |
| **Dexmethylphenidate** | - | Capsule (ER) | - | 0.182 |  | X applesauce (ER) | X food |  |
| **Dextroamphetamine and amphetamine** | - | Capsule, Tablet | - | - |  | X applesauce (capsule) | X Ora-sweet^®α^ |  |
| **Dextromethorphan** | - | Capsule, Liquid, Syrup | Weak base | 0.00851 |  |  | X meal |  |
| **Diazepam** | II (3) | Tablet, Solution | Weak base | 0.0122 |  |  | X food, water |  |
| **Diclofenac** | II (3) | Tablet, Capsule | Weak acid | 0.00447 | X milk |  | X food* |  |
| **Diltiazem** | I (3) | Capsule (ER) | Weak base | 0.0168 |  | X applesauce (ER capsules) |  |  |
| **Dimenhydrinate** | - | Tablet | Weak base | 0.00125 |  |  | X food |  |
| **Diphenhydramine** | - | Capsule, Solution, Syrup, Liquid | Weak base | 0.0752 |  |  | X meal |  |
| **Diphenoxylate and atropine** | - | Tablet, Solution | - | - |  |  | X food* |  |
| **Dipyridamole** | II (18) | Tablet, Solution | Weak base | 0.922 | X milk |  | X food*, cherry syrup, Ora-Sweet^®α^, Ora-Plus^®α^ (tablet) |  |
| **Docusate** | - | Liquid, Syrup | Weak acid | - | X milk, fruit juice, formula |  |  |  |
| **Dolasetron** | I (3) | Ampoule, Tablet | Weak base | 0.261 | X apple, apple-grape juice (solution) |  | X food (tablet) |  |
| **Doxazosin** | I (3) | Tablet | Weak base | 0.79 |  |  | X food, meal |  |
| **Doxepin** | I (19) | Solution | Weak base | 0.0319 | X water, milk, juice: orange, grapefruit, tomato, prune, pineapple juice |  |  | Do not mix with carbonated beverages |
| **Doxycycline** | IV (3) | Pellets DR, Capsule, Tablet | Amphoteric | 0.63 | X fluid. Avoid formula, milk, dairy products | X applesauce (pellets) | X food* |  |
| **Dronabinol** | - | Capsule | - | 2.8 |  |  | X meal |  |
| **Efavirenz** | II (4) | Capsule | Weak acid | 0.00855 | X formula | X^1^ |  | ^1^*e.g.* applesauce, grape jelly, yoghurt |
| **Elvitegravir, cobicistat, emtricitabine, and tenofovir alafenamide** | - | Tablet | - | - |  |  | X food |  |
| **Elvitegravir, cobicistat, emtricitabine, and tenofovir disoproxil fumarate** | - | Tablet | - | - |  |  | X food |  |
| **Emtricitabine** | I (20) | Capsule, Solution | Weak base | 112 |  |  | X food |  |
| **Emtricitabine, rilpivirine, and tenofovir alafenamide** | - | Tablet | - | - |  |  | X meal |  |
| **Emtricitabine and Tenofovir disoproxil fumarate** | - | Tablet | - | - |  |  | X food |  |
| **Emtricitabine, rilpivirine and tenofovir disoproxil fumarate** | - | Tablet | - | - |  |  | X meal (> 500 kcal) |  |
| **Enalapril** | I (3) | Tablet, Solution | Amphoteric | 16.4 |  |  | X food |  |
| **Ephedrine** | I (19) | Capsule | Weak base | 63.6 |  |  | X food |  |
| **Ergocalciferol** | III (13) | Capsule, Solution, Tablet | Neutral | 0.05 |  |  | X meal |  |
| **Ergotamine** | I (21) | Sublingual tablet | Amphoteric | 0.223 |  |  | X meal |  |
| **Ergotamine and caffeine** | - | Tablet | - | - |  |  | X meal |  |
| **Escitalopram** | I (22) | Solution, Tablet | - | 0.00588 |  |  | X food |  |
| **Esomeprazole** | - | Capsule | Amphoteric | 0.353 |  | X applesauce |  |  |
| **Estradiol** | I (13) | Tablet | Weak acid | 0.0213 |  |  | X food, meal* |  |
| **Estrogens** | - | Tablet | Weak acid | - |  |  | X food* |  |
| **Ethacrynic acid** | - | Tablet | Weak acid | 0.0194 | X milk |  | X food |  |
| **Ethambutol** | III (23) | Tablet | Weak base | 7.58 | X apple juice | X applesauce | X food | Do not mix with other juices or syrups (not stable) |
| **Ethionamide** | II (24) | Tablet | Amphoteric | 0.839 |  |  | X meal* |  |
| **Ethosuximide** | III (3) | Capsule,  Solution | Weak acid | 101 | X milk |  | X food* |  |
| **Etodolac** | II (7) | Capsule, Tablet | Weak acid | 0.0392 |  |  | X food* |  |
| **Etoposide** | II (7) | Ampoule | Weak acid | 0.978 | X orange or apple juice, lemonade |  |  |  |
| **Etravirine** | - | Tablet | - | 0.0169 | X water, milk, orange juice |  | X meal | Not grapefruit juice or carbonated beverages |
| **Everolimus** | IV(3) | Tablet | - | 0.00163 |  |  | X food |  |
| **Ezetimibe** | II (3) | Tablet | Neutral | 0.00846 |  |  | X meal |  |
| **Ezetimibe and Simvastatin** | - | Tablet | - | - |  |  | X meal |  |
| **Famciclovir** | III (3) | Tablet | Weak base | - |  |  | X food* |  |
| **Famotidine** | IV (3) | Solution, Tablet, Suspension | Amphoteric | 0.271 |  |  | X food, antiacids, Ora-Sweet^®α^, Ora-Plus^®α^ |  |
| **Felbamate** | II (7) | Tablet, Suspension | Weak base | 0.742 |  |  | X meal |  |
| **Felodipine** | II (7) | Tablet | Weak base | 0.00715 |  |  | X light meal |  |
| **Ferrous gluconate** | - | Tablet | - | - | X water, juice |  | X food* | Do not administer with milk products |
| **Ferrous sulfate** | - | Tablet, Syrup, Solution | - | - | X water, juice |  | X food* | Do not administer with milk or milk products |
| **Fexofenadine** | I (3) | Tablet, Suspension | Amphoteric | 0.00266 |  |  | X food | Avoid administration with fruit juices |
| **Flecainide** | I (22) | Tablet | Amphoteric | 0.0324 |  |  | X food | Avoid administration with milk or formulas |
| **Fluconazole** | III (3) | Tablet, Suspension | Weak base | 0.001 |  |  | X meal |  |
| **Fluoxetine** | I (3) | Capsule, Tablet, Solution | Weak base | 50 |  |  | X food |  |
| **Flurazepam** | - | Capsule | Weak base | 500 |  |  | X meal |  |
| **Flurbiprofen** | II (3) | Tablet | Weak acid | 0.0249 | X milk |  | X food, antacids* |  |
| **Fluvastatin** | II (25) | Capsule, Tablet | Weak acid | 0.00046 |  |  | X meal |  |
| **Fluvoxamine** | I (3) | Capsule, Tablet | Weak acid | 0.00734 |  |  | X meal |  |
| **Folic acid** | III (13) | Capsule, Tablet | Amphoteric | 0.0016 |  |  | X meal |  |
| **Fosamprenavir** | II (4) | Tablet, Suspension | Amphoteric | 0.685 |  |  | X food* | *food in paediatric patients and no food in adults (suspension) |
| **Fosinopril** | - | Tablet | Weak acid | 0.00101 |  |  | X food |  |
| **Furosemide** | IV (3) | Tablet | Weak acid | 0.118 | X milk |  | X food |  |
| **Gabapentin** | III (4) | Capsule | Amphoteric | 4.34 | X^1^ | X^2^ |  | ^1^*e.g.* orange juice  ^2^*e.g.* applesauce |
| **Glycopyrrolate** | - | Tablet | Weak base | 0.000944 |  |  | X meal |  |
| **Griseofulvin** | II (3) | Tablet, Suspension | Neutral | 0.0504 | X milk* | X applesauce, peanut butter or ice cream*; | X food*, meal (fatty) |  |
| **Guaifenesin** | - | Tablet, Liquid | Neutral | 14.9 | X fluid (large amount) |  |  |  |
| **Guaifenesin and codeine** | - | Tablet, Liquid, Capsule | - | - | X fluid (large amount) |  | X food* |  |
| **Guaifenesin and dextromethorphan** | - | Tablet, Liquid, Syrup | - | - | X fluid (large amount) |  |  |  |
| **Guanfacine** | I (13) | Tablet | Weak base | 0.139 | X water, milk or other liquid |  |  | Do not administer with high fat meal |
| **Haloperidol** | II (3) | Tablet, Solution | Amphoteric | 0.00446 | X water, acidic beverage,  milk* |  | X food * | Do not mix with coffee or tea |
| **Hydralazine** | III (3) | Tablet | Weak base | 2.61 |  |  | X food |  |
| **Hydro-chlorothiazide** | III (26) | Tablet, Capsule | Weak acid | 2.24 | X milk |  | X food |  |
| **Hydrocodone and acetaminophen** | - | Tablet, Capsule, Solution | - | - | X milk |  | X food* |  |
| **Hydrocodone and chlorpheniramine** | - | Capsule | - | - |  |  | X meal |  |
| **Hydrocortisone** | - | Tablet | Neutral | 0.199 | X milk |  | X food* |  |
| **Hydromorphone** | - | Capsule | Amphoteric | 4.39 | X milk (IR) |  | X food (IR)* |  |
| **Hydroxyzine** | II (3) | Tablet, Capsule, Solution, Syrup | Weak base | 700 |  |  | X food |  |
| **Hyoscyamine, atropine, scopolamine and phenobarbital** | - | Tablet | - | - |  |  | X meal |  |
| **Ibuprofen** | II (3) | Tablet, Capsule, Suspension | Weak acid | 0.0684 | X milk |  | X food* |  |
| **Imatinib** | II (4) | Tablet | Weak base | 0.0146 | X water, apple juice^1^ |  | X meal | ^1^50 mL -100 mg tablet  200 mL -400 mg tablet |
| **Imipramine** | I (13) | Tablet, Capsule | Weak base | 0.0182 |  |  | X food* |  |
| **Indinavir** | IV (26) | Capsule | Amphoteric | 0.015 | X water, liquids^1^ |  | X light snack^2^ | ^1^*e.g.* skim milk, coffee, tea, juice  ^2^ *e.g.* dry jelly toast, cornflakes w/ skim milk |
| **Indomethacin** | II (7) | Capsule, Suspension | Weak acid | 0.937 | X milk |  | X food, antacids* |  |
| **Iodoquinol** | - | Tablets | Amphoteric | 0.0815 |  | X applesauce | X chocolate syrup |  |
| **Irbesartan** | II (3) | Capsule | Amphoteric | 0.00884 |  | X applesauce (capsules) | X food |  |
| **Irinotecan** | - | Ampoule | Amphoteric | 0.11 | X cranberry grape juice |  |  |  |
| **Isotretinoin** | II (3) | Capsule | Weak acid | 0.00477 | X liquid |  | X meal |  |
| **Isradipine** | - | Capsule, Tablet | Weak base | 0.23 |  |  | X meal |  |
| **Itraconazole** | II (3) | Capsule | Weak base | 0.00964 |  |  | X food |  |
| **Ivacaftor** | - | Granules | - | 0.002 | X water, milk, juice | X^1^ |  | ^1^*e.g.* pureed fruit/vegetable, applesauce, yoghurt  Mix should be consumed within 1 hour |
| **Ketamine** | I (27) | Ampoule | Weak base | 0.046 | X cola, cherry juice, other beverages |  |  |  |
| **Ketoconazole** | II (7) | Tablet | Weak base | 8.66*10^-5^ | X acidic liquid^1^ |  |  | ^1^*e.g.* soda pop |
| **Ketorolac** | I (19) | Tablet | Weak acid | 2.5 (salt) | X milk |  | X food* |  |
| **Labetalol** | I (13) | Tablet | Amphoteric | 0.117 |  |  | X meal, cherry syrup |  |
| **Lacosamide** | I (28) | Solution, Tablet | - | 0.465 |  |  | X food |  |
| **Lactobacillus** | - | Granules, Capsules, Powder | - | - | X milk, fruit juice, water |  | X cereal, food |  |
| **Lactulose** | II (4) | Solution | Weak acid | 792 | X milk, fruit juice, water |  |  |  |
| **Lamivudine** | III (3) | Tablet | Weak base | 70 |  |  | X meal |  |
| **Lamivudine and Zidovudine** | - | Tablet | - | - |  |  | X meal |  |
| **Lamotrigine** | II (3) | Dispersible tablet | Weak base | 0.488 | X water, diluted fruit juice |  | X meals |  |
| **Lansoprazole** | II (7) | Capsule | Amphoteric | 0.00097 | X apple juice, orange juice, tomato juice^1^ | X applesauce, ensure pudding, cottage cheese, yoghurt or strained pears^2^ |  | ^1^60 mL  ^2^1 *tbs* |
| **Letrozole** | I (29) | Tablet | Weak base | 0.0799 |  |  | X meal |  |
| **Levetiracetam** | III (3) | Tablet | Neutral | 298 | X liquid |  |  |  |
| **Levocarnitine** | - | Solution | Amphoteric | 2500 | X beverages, liquid food |  |  |  |
| **Levocetirizine** | III (22) | Tablet, Solution | Amphoteric | 0.0658 |  |  | X food |  |
| **Levofloxacin** | I (3) | Tablet | Amphoteric | 1.44 |  |  | X food |  |
| **Levothyroxine** | I (3) | Tablet | Amphoteric | 0.105 | X breast milk, formula^1^, water* |  | X food | ^1^non-soy |
| **Linezolid** | IV (3) | Tablet, Suspension | Amphoteric | 3 |  |  | X food |  |
| **Lisdexamfetamine** | - | Capsule | - | 792 |  |  | X food |  |
| **Lisinopril** | III (13) | Tablet | Amphoteric | 97 |  |  | X food |  |
| **Lithium** | I (26) | Tablet, Suspension, Capsule | Weak acid | - |  |  | X meal* |  |
| **Lopinavir and ritonavir** | - | Tablet, Solution | - | - |  |  | X food^1^ | ^1^Adm with sweet foods, chocolate syrup, or peanut butter to help mask taste |
| **Loratadine** | II (15) | Tablet, Capsule, Syrup, Solution | Weak base | 0.000011 |  |  | X meal |  |
| **Lorazepam** | I (22) | Solution | Amphoteric | 0.08 | X water, juice, soda | X^1^ | X food* | ^1^ *e.g.* applesauce, pudding |
| **Losartan** | I (13) | Tablet | Amphoteric | 0.00082 |  |  | X food |  |
| **Lumacaftor and ivacaftor** | - | Tablet | - | - |  |  | X high-fat food^1^ | ^1^*e.g.* eggs, avocados, nuts, butter, peanut butter, cheese pizza, whole milk dairy products |
| **Magnesium-aspartate hydrochloride** | - | Granules | - | - | X water, juice |  |  |  |
| **Magnesium sulfate** | - | Granules | - | 710 | X lemon juice |  |  |  |
| **Maraviroc** | III (30) | Tablet | Weak base | 0.00883 |  |  | X meal |  |
| **Mebendazole** | II (3) | Tablet | Amphoteric | 0.0713 |  |  | X food |  |
| **Meclizine** | II (22) | Tablet | Weak base | 0.001 |  |  | X food* |  |
| **Medium chain triglycerides** | - | Emulsion oil | - | - | X water, other beverage (*e.g.* juice, milk) |  | X sauces, salad dressings, other foods |  |
| **Medroxy-progesterone** | IV (20) | Tablet | Neutral | 0.0021 |  |  | X food |  |
| **Mefloquine** | II (4) | Tablet | Amphoteric | 0.038 | X water, milk, chocolate syrup | X applesauce, jelly | X food |  |
| **Megestrol** | II (25) | Suspension | Neutral | 0.00336 |  |  | X food |  |
| **Meloxicam** | IV (22) | Tablet, Capsule, Suspension | Weak acid | 0.00715 | X milk |  | X food* |  |
| **Mesalamine** | IV (4) | Capsule | Amphoteric | 0.84 |  | X yoghurt or peanut butter^1^ | X food with a pH < 6 | ^1^Pentasa^®^ |
| **Metformin** | III (4) | Tablet | Weak base | 1.38 |  |  | X meal* |  |
| **Methadone** | I (31) | Tablet, Solution | Weak base | 0.0059 | X juice, water |  |  |  |
| **Methenamine** | - | Tablet | Weak base | 766 | X cranberry juice* |  | X food* |  |
| **Methsuximide** | - | Capsule | Neutral | 2.13 |  |  | X food |  |
| **Methyldopa** | III (3) | Tablet | Amphoteric | 10 |  |  | X food |  |
| **Methylphenidate** | I (4) | Tablet, Capsule | Weak base | 1.25 | X water, milk or juice ^1^ | X applesauce ^2^ |  | ^1^Concerta^®^, Metadate^®^ ^2^Metadate^®^, Ritaline LA^®^ |
| **Methylprednisolone** | - | Tablet | Weak acid | 0.120 | X milk |  | X food | Not grapefruit juice |
| **Metolazone** | - | Tablet | Weak acid | 0.06 |  |  | X food*, cherry syrup, Ora-Sweet^®α^, Ora-Plus^®α^ |  |
| **Metronidazole** | IV (3) | Tablet  Capsule | Weak base | 4.5 |  |  | X food*, Ora-Sweet^®α^, Ora-Plus^®α^ |  |
| **Metyrapone** | - | Capsule | Weak base | 0.427 | X milk * | Yoghurt | X food* |  |
| **Mexiletine** | I (3) | Capsule | Weak base | 8.25 | X milk |  | X food* |  |
| **Mineral oil** | - | Suspension | - | Not soluble | X milk, water |  | X cocoa |  |
| **Minoxidil** | III (13) | Tablet | Weak base | 2.2 |  |  | X food |  |
| **Mitotane** | - | Tablet | Neutral | 0.0001 |  |  | X MCT oil, then solution mixed with a fatty food (milk or yoghurt) |  |
| **Modafinil** | IV (3) | Tablet | Neutral | 0.622 |  |  | X food |  |
| **Montelukast** | II (4) | Granules | Amphoteric | 8.2*10^-6^ | X baby formula or breast milk^1^ | X applesauce, ice cream^2^ | X rice, carrots | ^1^ 5 mL  ^2^ a spoonful  Cold or at room temp. |
| **Morphine** | I (4) | Capsule (ER) | Amphoteric | 0.149 |  | X applesauce | X food |  |
| **Nadolol** | III (19) | Tablet | Weak base | 8.33 |  |  | X meal |  |
| **Naproxen** | II (7) | Tablet, Suspension | Weak acid | 0.0159 | X milk |  | X food, antacids* |  |
| **Nefazodone** | - | Tablet | Weak base | 0.0698 |  |  | X food |  |
| **Nelfinavir** | II (3) | Tablet | Amphoteric | 0.00191 |  |  | X food |  |
| **Neostigmine** | I (3) | Tablet | Weak base | 0.0677 |  |  | X food |  |
| **Nevirapine** | II (3) | Tablet | Weak base | 0.0007 | X milk, water or soda (IR) |  | X meal |  |
| **Niacin** | III (3) | Tablet, Capsule, Powder | Amphoteric | 18 | X milk |  | X food*, low fat snack |  |
| **Nicardipine** | I (3) | Capsule | Weak base | 0.0022 |  |  | X meals | Avoid high fat meals |
| **Nifedipine** | II (3) | Capsule | Weak base | 0.0177 |  |  | X food |  |
| **Nitisinone** | - | Capsule | Weak acid | 0.00811 | X water, formula | X applesauce |  |  |
| **Nitrofurantoin** | IV (3) | Suspension | Weak base | 0.0795 | X water, milk*, fruit juice, or formula |  | X food |  |
| **Nizatidine** | III (3) | Tablet, Capsule | Weak base | 0.039 | X ^1^  (capsule) |  | X food (tablet) | ^1^ Lemon-lime Gatorade^®^, Ocean- Spray^®^ Cran-Grape juice, V8^®^100% vegetable juice  Do not administer or mix with apple juice |
| **Norethindrone** | I (3) | Tablet | Neutral | 0.0069 |  |  | X food |  |
| **Nortriptyline** | I (22) | Capsule, Solution | Weak base | 0.00087 |  |  | X food |  |
| **Olanzapine** | I (4) | Tablet | Weak base | 0.0942 |  |  | X food |  |
| **Olmesartan** | II (32) | Tablet | Weak base | 0.0105 |  |  | X food |  |
| **Omeprazole** | II (4) | Capsule | Amphoteric | 0.359 |  | X applesauce |  |  |
| **Ondansetron** | I (3) | Tablet, Solution | Weak base | 0.248 |  |  | X food, cherry syrup, Ora-Sweet^®α^, Ora-Plus^®α^ |  |
| **Oseltamivir** | III (3) | Capsule | Amphoteric | 0.686 | X food, sweetened liquid ^1^ |  |  | ^1^*e.g.* chocolate syrup, corn syrup, caramel topping, light brown sugar dissolved in water |
| **Oxaprozin** | II (7) | Tablet | Weak acid | 0.0325 | X milk |  | X food* |  |
| **Oxcarbazepine** | IV (3) | Tablet, Suspension | Neutral | 0.308 |  |  | X meal |  |
| **Oxybutynin** | I (3) | Tablet, Syrup,  Capsule | Amphoteric | 0.01 | X liquid (capsules) |  | X food |  |
| **Oxycodone** | IV (3) | Tablet, Capsule, Solution | Amphoteric | 5.59 |  |  | X food* |  |
| **Oxycodone and acetaminophen** | - | Tablet, Capsule, Solution | - | - | X milk |  | X food* |  |
| **Oxycodone and aspirin** | - | Tablet | - | - | X milk |  | X food* |  |
| **Paliperidone** | II (33) | Tablet | Weak base | 0.297 | X liquids (ER tablets) |  |  |  |
| **Pancrelipase** | - | Tablet, Capsule | - | 1 | X liquid, water, or juice | X small amount of acidic food (pH ≤ 4.5) ^1^ | X snacks, meal | ^1^*e.g.* applesauce, prepared baby food  Infants: avoid mixing with breast milk or formula. |
| **Pantoprazole** | III (22) | Suspension (DR) | Amphoteric | 0.495 | X apple juice^1^ | X applesauce^2^ |  | ^1^5 mL; ^2^1 tbs  Do not administer with other liquids or foods |
| **Paricalcitol** | - | Capsule | Neutral | 0.0068 |  |  | X food |  |
| **Paromomycin** | - | Capsule | Amphoteric | 79.7 |  |  | X meal |  |
| **Paroxetine** | I (3) | Capsule, Tablet, Suspension | Weak base | 0.00853 |  |  | X meal* |  |
| **Penicillamine** | III (3) | Capsule | Amphoteric | 111 | X fruit juice | X chilled puree fruit |  |  |
| **Penicillin V Potassium** | III (19) | Tablet, Solution | Weak acid | 0.454 |  |  | X food* |  |
| **Pentobarbital** | - | Ampoule | Weak acid | 6.78 |  |  | X cherry syrup |  |
| **Pentoxifylline** | - | Tablet | Neutral | 77 |  |  | X food or antacids* |  |
| **Perampanel** | - | Tablet, Suspension | - | 0.0056 |  |  | X meal |  |
| **Perphenazine** | - | Tablet | Weak base | 0.0283 |  |  | X meal* |  |
| **Phenazopyridine** | II (7) | Tablet | Weak base | 0.202 |  |  | X food* |  |
| **Phenobarbital** | I (19) | Solution | Weak acid | 1.1 | X water, milk or juice |  |  |  |
| **Phenoxybenzamine** | - | Capsule | Weak base | 0.0103 | X milk* |  |  |  |
| **Phenytoin** | II (3) | Capsule (ER) | Weak acid | 0.032 |  |  | X meal |  |
| **Phytonadione** | - | Tablet | Neutral | 5.92e05 |  |  | X food |  |
| **Pimozide** | II (3) | Tablet | Amphoteric | 0.01 |  |  | X meal |  |
| **Piroxicam** | II (7) | Capsule | Amphoteric | 0.023 | X milk |  | X food* |  |
| **Posaconazole** | II (34) | Tablet | Amphoteric | 0.012 |  |  | X food (DR tablets) |  |
| **Potassium chloride** | I (22) | Powder, Capsule, Solution | Neutral | 357 | X dilute in 6 ounces of water, juice or another beverage (powder)  Juice, water (solution) | X applesauce or pudding^1^ (capsule) |  | ^1^1 tbs |
| **Potassium citrate and citric acid** | - | Powder | - | - | X dilute in at least 6 ounces of water or juice |  |  |  |
| **Potassium iodide** | I (4) | Solution | Neutral | 1428.6 | X ^1^:water, milk, broth, fruit juice;  formula, soda, orange juice, milk |  | X^2^: Raspberry syrup | ^1^ SSKI^®^  ^2^ Iosat^®^, Thyrosafe^®^ |
| **Potassium iodide and iodine** | - | Solution | - | - | X water, fruit juice or milk |  |  |  |
| **Potassium phosphate and sodium phosphate** | - | Tablet  Powder | - | - | X juice (powder) |  | X food (tablet) |  |
| **Pravastatin** | III (3) | Tablet | Weak acid | 0.242 |  |  | X meal |  |
| **Prazosin** | I (26) | Capsule | Weak base | 0.5 |  |  | X meal |  |
| **Prednisolone** | I (35) | Tablet, Solution, Suspension | Weak acid | 0.223 | X milk |  | X food, meal* |  |
| **Prednisone** | I (36) | Tablet, Solution | Weak acid | 0.111 | X milk |  | X food* |  |
| **Primaquine** | I (21) | Tablet | Weak base | 0.0564 |  |  | X food* |  |
| **Primidone** | II (7) | Tablet | Weak acid | 0.5 |  |  | X food* |  |
| **Probenecid** | - | Tablet | Weak acid | 0.021 |  |  | X food* |  |
| **Prochlorperazine** | II (21) | Tablet | Weak base | 0.015 | X water |  | X food |  |
| **Promethazine** | I (26) | Tablet, Syrup, Solution | Weak base | 0.0156 | X milk, water |  | X food* |  |
| **Promethazine and phenylephrine** | - | Syrup | - | - | X milk, water |  | X food |  |
| **Promethazine, phenylephrine and codeine** | - | Syrup | - | - | X water |  | X food* |  |
| **Propranolol** | I (3) | Solution, Capsule | Neutral | 0.0617 | X water, fruit juice, liquid (oral solution) | X semi solid food (oral solution) | X food (ER capsules) |  |
| **Protriptyline** | - | Tablet | Weak base | 0.00104 |  |  | X food* | Do not administer with grapefruit juice |
| **Pseudoephedrine** | III (3) | Tablet, Syrup, Liquid | Amphoteric | 0.007 | X milk*, water |  |  |  |
| **Pseudoephedrine and ibuprofen** | - | Tablet, Capsule | - | - |  |  | X food |  |
| **Psyllium** | - | Granules, Powder | - | - | X water or juice^1^ |  |  | ^1^ 8 ounces |
| **Pyrantel pamoate** | - | Tablet | Weak acid | 0.118 | X milk or fruit juice |  |  |  |
| **Pyridoxine** | III (3) | Capsule, Tablet | Amphoteric | 220 |  |  | X meal |  |
| **Pyrimethamine** | IV (26) | Tablet | Weak base | 0.121 |  |  | X meal |  |
| **Quetiapine** | II (3) | Tablet | Weak base | 0.0403 |  |  | X light meal |  |
| **Quinapril** | I (3) | Tablet | Amphoteric | 0.001 |  |  | X food |  |
| **Quinidine** | I (26) | Tablet | Amphoteric | 0.140 | X milk |  | X food*, cherry syrup, Ora-Sweet^®α^, Ora-Plus^®α^ |  |
| **Rabeprazole** | III (3) | Capsule | Amphoteric | 0.336 | X liquid | X |  | ^1^ *e.g.* formula, apple juice, paediatric electrolyte solution (small amount)  ^2^ *e.g.* applesauce, fruit or vegetable-based baby food, yoghurt (small amounts) |
| **Raltegravir** | II (37) | Tablet | Weak acid | - |  |  | X meal |  |
| **Ranitidine** | III (3) | Tablet, Syrup, Capsule | Weak base | 24.7 |  |  | X food, meal |  |
| **Ribavirin** | III (3) | Tablet, Capsule,  Solution | Neutral | 33.2 |  |  | X food |  |
| **Riboflavin** | I (26) | Tablet, Capsule | Weak acid | 0.0847 |  |  | X food |  |
| **Rifabutin** | II (38) | Capsule | Amphoteric | 0.19 |  | X applesauce |  |  |
| **Rifampicin** | II (3) | Capsule | Amphoteric | 1.4 |  | X applesauce or jelly |  |  |
| **Rifaximin** | IV (39) | Tablet | Amphoteric | 0.00738 |  |  | X food |  |
| **Rimantadine** | - | Tablet | Weak base | 50 (hydro-chloride salt) |  |  | X food |  |
| **Risperidone** | II (4) | Solution | Weak base | 2.33 | X water, coffee, orange juice, or low-fat milk |  |  | Do not mix with coffee or tea |
| **Ritonavir** | IV (4) | Liquid | Amphoteric | 0.00126 | X milk, chocolate milk | X vanilla/ chocolate pudding, ice cream | X nutritional supplement | Other techniques: dulling the taste buds by chewing ice, giving popsicles of partially frozen orange or grape fruit concentrates, coating the mouth with peanut butter, administration of strong-tasting foods immediately after a dose. |
| **Rosiglitazone** | I (3) | Tablet | Amphoteric | 0.038 |  |  | X meal |  |
| **Rosuvastatin** | III (40) | Tablet | Amphoteric | 0.0886 |  |  | X food |  |
| **Rufinamide** | II (41) | Tablet | Neutral | 0.642 |  |  | X food |  |
| **Sacrosidase** | - | Solution | - | - | X water, milk, formula |  |  |  |
| **Sapropterin** | III (42) | Powder, Tablet | Amphoteric | 20  (dichloride salt) | X water, apple juice, formula | X (powder) |  | ^2^applesauce or pudding (small amount) |
| **Saquinavir** | I (3) | Capsule | Amphoteric | 0.00765 |  | X jam^2^ | X sugar or sorbitol syrup | ^1^ 3 teaspoons  ^2^ 15 mL |
| **Senna** | - | Syrup | - | - | X juice or milk | X ice cream |  |  |
| **Sertraline** | I (4) | Solution | Weak base | 0.0035 | X water, orange juice, lemonade, ginger ale or lemon/lime soda |  |  | Do not administer with grapefruit juice |
| **Sildenafil** | I (3) | Tablet, Suspension | Amphoteric | 3.5 |  |  | X meal |  |
| **Simethicone** | - | Tablet, Suspension, Capsule | - | 1.71 | X water, formula, liquids |  |  |  |
| **Simvastatin** | II (43) | Tablet | Neutral | 0.0122 |  |  | X meal |  |
| **Sirolimus** | II (4) | Solution | Weak acid | 0.00173 | X water, orange juice |  |  |  |
| **Sodium phenylbutyrate** | - | Powder | - | 18 | X |  | X food, meal or feeding | Avoid mixing with acidic beverages |
| **Sodium phosphate** | - | Tablet | - | 121 | X clear liquids^1^ |  |  | ^1^ water, flavoured water, pulp-free lemonade, ginger ale or apple juice (8 ounces) |
| **Sodium polystyrene sulfonate** | - | Powder | - | - |  |  | X syrup | Do not mix with orange juice |
| **Sotalol** | I (13) | Solution, Tablet | Amphoteric | 5.51 |  |  | X meal |  |
| **Spironolactone** | II (3) | Tablet | Neutral | 0.022 |  |  | X food |  |
| **Stavudine** | I (26) | Capsule | Weak acid | 40.5 |  |  | X food |  |
| **Succimer** | - | Capsule | Weak acid | 2.43 | X fruit juice | X^1^ |  | ^1^small amount |
| **Sulfamethoxazole and trimethoprim** | - | Tablet, Suspension | - | - |  |  | X meal |  |
| **Sulindac** | - | Tablet | Weak acid | 3 | X milk |  | X food |  |
| **Sumatriptan** | III (3) | Tablet | Amphoteric | 21.4 | X water, other fluids |  |  |  |
| **Tamoxifen** | II (3) | Tablet, Solution | Weak base | 0.00102 |  |  | X food |  |
| **Tamsulosin** | I (3) | Capsule | Amphoteric | 0.0066 | X juice | X^1^ |  | ^1^yoghurt or pudding |
| **Temozolomide** | I (3) | Capsule | Weak base | 5.09 | X apple juice | X applesauce |  |  |
| **Tenofovir disoproxil fumarate** | III (4) | Powder | Weak base | 13.4 |  | X applesauce, baby food, yoghurt ^1^ | X food | ^1^ 2-4 ounces  Do not mix with liquids |
| **Terazosin** | - | Capsule | Weak base | 29.7 |  |  | X meal |  |
| **Terbinafine** | I (3) | Granules | Weak base | 0.0007 |  | X non-acidic food ^1^ |  | ^1^ pudding, mashed potatoes. Do not use fruit-based foods |
| **Terbutaline** | - | Tablet | Amphoteric | 213 |  |  | X meal |  |
| **Theophylline** | I (4) | Capsule | Weak base | 7.36 |  | X |  |  |
| **Thiamine** | III (3) | Tablet, Capsule | Weak base | 500 |  |  | X food |  |
| **Thioridazine** | - | Solution, Tablet | Weak base | 3.36*10^-5^ | X water*^1^, milk*, juice^1^ |  | X food* | ^1^oral concentrate |
| **Thiothixene** | - | Capsule | Weak base | 0.0139 | X water |  | X food |  |
| **Tiagabine** | - | Tablet | Amphoteric | 0.0211 |  |  | X food |  |
| **Tinidazole** | - | Tablet | Weak base | 3.03 |  |  | X food*, cherry syrup |  |
| **Tolmetin** | - | Tablet, Capsule | Weak acid | 0.222 | X milk |  | X food, antacids* |  |
| **Tolterodine** | I (3) | Tablet, Capsule | Amphoteric | 0.00534 |  |  | X food |  |
| **Topiramate** | III (4) | Capsule | Weak acid | 9.8 |  | X applesauce, ice cream, pudding, custard, yoghurt, or oatmeal |  | ^1^ 1 *tbs* |
| **Topotecan** | - | Capsule, Ampoule | Amphoteric | 1 | X acidic medium^1^ |  |  | ^1^*e.g.* apple, grape or orange juice (30 mL) |
| **Torsemide** | - | Tablet | Weak acid | 0.0596 |  |  | X meal |  |
| **Tramadol** | I (3) | Capsule, Tablet, Suspension | Weak base | 0.75 |  |  | X food |  |
| **Tranexamic acid** | I (44) | Tablet | Amphoteric | 167 |  |  | X meal |  |
| **Tretinoin** | - | Capsule | Weak acid | 0.0048 | X warm milk | X^1^ | X meal | ^1^one spoonful |
| **Triamterene** | II (3) | Capsule | Weak base | 0.0482 |  |  | X food* |  |
| **Trifluoperazine** | - | Tablet | Weak base | 0.0122 |  |  | X food* |  |
| **Trimethobenzamide** | - | Capsule | Weak base | 0.04 |  |  | X food |  |
| **Trimethoprim** | IV (3) | Tablet, Solution | Weak base | 0.400 | X milk |  | X food* |  |
| **Ursodiol** | II (43) | Tablet, Capsule | Weak acid | 0.02 |  |  | X food, Ora-Sweet^®α^, Ora-Plus^®α^ |  |
| **Valacyclovir** | III (43) | Tablet | Amphoteric | 3.55 |  |  | X food |  |
| **Valganciclovir** | III (45) | Tablet | Amphoteric | 4.79 |  |  | X meal |  |
| **Valproic acid and derivatives** | II (3) | Capsule | Weak acid | 1.3 (valproic acid) |  | X^1^ | X food* | ^1^*e.g.* pudding, applesauce (small amount)  Do not administer with carbonated drinks |
| **Valsartan** | II (3) | Tablet | Weak acid | 0.0234 |  |  | X food |  |
| **Vancomycin** | - | Solution | Amphoteric | 0.225 |  |  | X flavouring syrup |  |
| **Venlafaxine** | I (3) | Tablet, Capsule | Weak base | 572 (hydrochloride salt) |  | X applesauce (capsule) | X food |  |
| **Verapamil** | II (3) | Capsule | Weak base | 0.00447 |  | X applesauce^1^ (capsules^2^) | X food (tablet, caps), cherry syrup, Ora-Sweet^®α^, Ora-Plus^®α^ | ^1^1 *tbs*  ^2^only 2 of the commercial formulations |
| **Vigabatrin** | I (4) | Tablet | Amphoteric | 55.1 |  |  | X food |  |
| **Vitamin A** | - | Capsule | Neutral | 0.00067 | X milk |  | X food |  |
| **Vitamin E** | - | Capsule, Tablet, Solution | Neutral | 7.04E^-6^ |  |  | X food |  |
| **Warfarin** | I (46) | Tablet | Weak acid | 0.017 |  |  | X food |  |
| **Zinc sulfate** | - | Capsule, Tablet | - | - |  |  | X food* |  |
| **Ziprasidone** | II (47) | Capsule | Weak base | 0.00718 |  |  | X food |  |
| **Zonisamide** | - | Capsule | Weak acid | 0.8 |  |  | X meal |  |

■ Drug included in the UK formularies but without recommendations of medicine co-administration with vehicles

■ Drug included in both the UK formularies and the Lexicomp Handbook with recommendations for medicine co-administration with vehicles

(underlined) Recommendations are to mix ‘*with or without food/meals’* or *‘without regards to food/meals’*

**^¥^** Calculated using Advanced Chemistry Development (ACD/Labs) Software V11.02 (© 1994-2016 ACD/Labs)

* To avoid GI distress α for extemporaneous preparations

ER: Extended release DR: Delayed release

Tbs: tablespoon mL: millilitre

**References**

1. Taketomo CK, Hodding JH, Kraus DM. Pediatric & neonatal dosage handbook: an extensive resource for clinicians treating pediatric and neonatal patients. 23 ed. Hudson, Ohio: Lexicomp; 2017.

2. Wishart DS, Knox C, Guo AC, Cheng D, Shrivastava S, Tzur D, et al. DrugBank: a knowledgebase for drugs, drug actions and drug targets. Nucleic Acids Res. 2008;36(suppl 1):D901-D6.

3. Drug Delivery Foundation: BCS database. 2015. http://www.tsrlinc.net/search.cfm. Assessed 5 November 2018.

4. Martir J, Flanagan T, Mann J, Fotaki N. Recommended strategies for the oral administration of paediatric medicines with food and drinks in the context of their biopharmaceutical properties: a review. J Pharm Pharmacol. 2017;69(4):384-97.

5. Dave VS, Gupta D, Yu M, Nguyen P, Varghese Gupta S. Current and evolving approaches for improving the oral permeability of BCS Class III or analogous molecules. Drug Dev Ind Pharm. 2017;43(2):177-89.

6. Informe Público de Evaluación: Almotriptan Cinfa 12.5 MG Comprimidos. 2013. https://cima.aemps.es/cima/pdfs/ipe/78686/IPE_78686.pdf [spanish]. Accessed 6 January 2019.

7. Selvamuthukumar S, Anandam S, Krishnamoorthy K, Rajappan M. Nanosponges: A novel class of drug delivery system-review. J Pharm Pharm Sci. 2012;15(1):103-11.

8. Thambavita D, Galappatthy P, Mannapperuma U, Jayakody L, Cristofoletti R, Abrahamsson B, et al. Biowaiver monograph for immediate-release solid oral dosage forms: amoxicillin trihydrate. J Pharm Sci. 2017;106(10):2930-45.

9. Georgaka D, Butler J, Kesisoglou F, Reppas C, Vertzoni M. Evaluation of dissolution in the lower intestine and its impact on the absorption process of high dose low solubility drugs. Mol Pharm. 2017;14(12):4181-91.

10. Bamigbola EA. Correlation of in vitro dissolution profiles with in vivo pharmacokinetic parameters of some commercial brands of aspirin tablets marketed in Nigeria. Readings in Advanced Pharmacokinetics-Theory, Methods and Applications: InTech; 2012.

11. European Medicines Agency: Assessment report Atazanavir Mylan. 2016. https://www.ema.europa.eu/documents/assessment-report/atazanavir-mylan-epar-public-assessment-report_en.pdf. Accessed 2 February 2019.

12. Smetanova L, Stetinova V, Kholova D, Kvetina J, Smetana J, Svoboda Z. Caco-2 cells and Biopharmaceutics Classification System (BCS) for prediction of transepithelial transport of xenobiotics (model drug: caffeine). Neuroendocrinol Lett. 2009;30:101-5.

13. Chatzizacharia K, Hatziavramidis D. New frames of reference for mapping drugs in the four classes of the BCS and BDDCS into regions with clear boundaries. AICHE J. 2015;61(11):3570-9.

14. Oral drug absorption: Prediction and assessment. Dressman JB, Lennernas H, editors. New York: CRC Press; 2000.

15. Shawahna R. Pediatric biopharmaceutical classification system: using age-appropriate initial gastric volume. AAPS J. 2016;18(3):728-36.

16. Sanphui P, Devi VK, Clara D, Malviya N, Ganguly S, Desiraju GR. Cocrystals of hydrochlorothiazide: solubility and diffusion/permeability enhancements through drug–coformer interactions. Mol Pharm. 2015;12(5):1615-22.

17. Papich MG, Martinez MN. Applying biopharmaceutical classification system (BCS) criteria to predict oral absorption of drugs in dogs: challenges and pitfalls. AAPS J. 2015;17(4):948-64.

18. Vora C, Patadia R, Mittal K, Mashru R. Preparation and characterization of dipyridamole solid dispersions for stabilization of supersaturation: effect of precipitation inhibitors type and molecular weight. Pharm Dev Technol. 2016;21(7):847-55.

19. Khojasteh SC, Wong H, Hop CECA. Oral Absorption. Drug Metabolism and Pharmacokinetics Quick Guide. New York, NY: Springer New York; 2011. p. 47-56.

20. Plöger GF, Hofsäss MA, Dressman JB. Solubility Determination of Active Pharmaceutical Ingredients Which Have Been Recently Added to the List of Essential Medicines in the Context of the Biopharmaceutics Classification System–Biowaiver. J Pharm Sci. 2018;107(6):1478-88.

21. Shawahna R, Rahman N. Evaluation of the use of partition coefficients and molecular surface properties as predictors of drug absorption: a provisional biopharmaceutical classification of the list of national essential medicines of Pakistan. Daru. 2011;19(2):83.

22. Intra-Agency Agreement Between the Eunice Kennedy Shriver National Institute of Child Health and Human Development (NICHD) and the U.S. Food and Drug Administration (FDA) Oral Formulations Platform—Report 1. 2011. http://bpca.nichd.nih.gov/collaborativeefforts/initiatives/upload/Formulations_Table_for_Web_11-02-11.pdf. Accessed 20 February 2016.

23. Becker C, Dressman J, Amidon G, Junginger H, Kopp S, Midha K, et al. Biowaiver monographs for immediate release solid oral dosage forms: Ethambutol dihydrochloride. J Pharm Sci. 2008;97(4):1350-60.

24. Nechipadappu SK, Trivedi DR. Pharmaceutical salts of ethionamide with GRAS counter ion donors to enhance the solubility. Eur J Pharm Sci. 2017;96:578-89.

25. Li Y, Song CK, Kim MK, Lim H, Shen Q, Lee DH, et al. Nanomemulsion of megestrol acetate for improved oral bioavailability and reduced food effect. Arch Pharm Res. 2015;38(10):1850-6.

26. World Health Organisation: Proposal to waive in vivo bioequivalence requirements for WHO Model List of Essential Medicines immediate-release, solid oral dosage forms (Annex 8) - WHO Technical Report Series, No. 937. 2006. http://apps.who.int/prequal/info_general/documents/TRS937/WHO_TRS_937__annex8_eng.pdf. Accessed 2 March 2016.

27. Clinical Pharmacology Review: Surge Dose^®^ ketamine as an oral analgesic and sedative agent. 2012. http://www.imaginot.com.au/downloads/d8a8979b-750a-45d0-bd31-c97a81bcd049.pdf. Accessed 2 February 2019.

28. European Medicines Agency: Assessment report for vimpat. 2008. https://www.ema.europa.eu/documents/assessment-report/vimpat-epar-public-assessment-report_en.pdf. Accessed 02 February 2019.

29. Australian Product Information: Apo-Letrozole (Letrozole). 2018. http://www.medicines.org.au/files/txpletro.pdf. Accessed 10 January 2019.

30. European Medicines Agency: Assessment report for celsentri. 2017. https://www.ema.europa.eu/documents/variation-report/celsentri-h-c-000811-x-0046-g-epar-assessment-report-variation_en.pdf. Accessed 5 January 2019.

31. Medicines Evaluation Board in the Netherlands: Public assessment report of methadone (as hydrochloride). 2010. https://db.cbg-meb.nl/Pars/h34508.pdf. Accessed 9 January 2019.

32. Thakkar HP, Patel BV, Thakkar SP. Development and characterization of nanosuspensions of olmesartan medoxomil for bioavailability enhancement. J Pharm Bioallied Sci. 2011;3(3):426.

33. Sherje AP, Londhe V. Ternary inclusion complex of paliperidone with β-cyclodextrin and hydrophilic polymer for solubility and dissolution enhancement. J Pharm Innov. 2015;10(4):324-34.

34. Gubbins PO, Krishna G, Sansone-Parsons A, Penzak SR, Dong L, Martinho M, et al. Pharmacokinetics and safety of oral posaconazole in neutropenic stem cell transplant recipients. Antimicrob Agents Chemother. 2006;50(6):1993-9.

35. Vogt M, Derendorf H, Krämer J, Junginger H, Midha K, Shah V, et al. Biowaiver Monographs for Immediate Release Solid OralDosage Forms: Prednisolone. J Pharm Sci. 2007;96(1):27-37.

36. Vogt M, Derendorf H, Kramer J, Junginger HE, Midha KK, Shah VP, et al. Biowaiver monographs for immediate release solid oral dosage forms: prednisone. J Pharm Sci. 2007;96(6):1480-9.

37. Date AA, Destache CJ. A review of nanotechnological approaches for the prophylaxis of HIV/AIDS. Biomaterials. 2013;34(26):6202-28.

38. Benet LZ, Broccatelli F, Oprea TI. BDDCS applied to over 900 drugs. AAPS J. 2011;13(4):519-47.

39. Danish M, Shetsandi A, Bhise KS. Formulation development and taste masking of rifaximin nanosuspension. Inventi Rapid: Pharm Tech. 2013.

40. Medicines & Healthcare products Regulatory Agency. Public assessment report decentralised procedure: Rosuvastatin. 2001. http://www.mhra.gov.uk/home/groups/par/documents/websiteresources/con744443.pdf. Accessed 11 January 2019.

41. Wandera OR. Formulation development of generic rufinamid uncoated tablets: University of Nairobi; 2015.

42. Australian Government Department of Health and Ageing: Australian Public Assessment Report for Sapropterin dihydrochloride. 2011. https://www.tga.gov.au/sites/default/files/auspar-kuvan.pdf. Accessed 7 January 2019.

43. Ono A, Tomono T, Ogihara T, Terada K, Sugano K. Investigation of biopharmaceutical and physicochemical drug properties suitable for orally disintegrating tablets. ADMET DMPK. 2016;4(4):335-60.

44. Visych SY, Dorovskyy O, Andryukova L, Fetisova O. Bioequivalence studies of medicinal products of tranexamic acid. Ukr bìofarm ž. 2016(6 (47)):24-8.

45. Chakraborty S, Yadav L, Aggarwal D. Prediction of in vivo drug performance using in vitro dissolution coupled with STELLA: a study with selected drug products. Drug Dev Ind Pharm. 2015;41(10):1667-73.

46. Endrenyi L, Tothfalusi L. Determination of bioequivalence for drugs with narrow therapeutic index: reduction of the regulatory burden. J Pharm Pharm Sci. 2013;16(5):676-82.

47. Zakowiecki D, Cal K, Kaminski K, Adrjanowicz K, Swinder L, Kaminska E, et al. The improvement of the dissolution rate of ziprasidone free base from solid oral formulations. AAPS PharmSciTech. 2015;16(4):922-33.
